# Modeling Fatty Acid Transfer from Artery to Cardiomyocyte

**DOI:** 10.1371/journal.pcbi.1004666

**Published:** 2015-12-16

**Authors:** Theo Arts, Robert S. Reneman, James B. Bassingthwaighte, Ger J. van der Vusse

**Affiliations:** 1 Department of Biomedical Engineering, CARIM, Maastricht University, Maastricht, the Netherlands; 2 Department of Physiology, CARIM, Maastricht University, Maastricht, the Netherlands; 3 Department of Bioengineering, University of Washington, Seattle, Washington, United States of America; Johns Hopkins University, UNITED STATES

## Abstract

Despite the importance of oxidation of blood-borne long-chain fatty acids (*Fa*) in the cardiomyocytes for contractile energy of the heart, the mechanisms underlying the transfer of *Fa* from the coronary plasma to the cardiomyocyte is still incompletely understood. To obtain detailed insight into this transfer process, we designed a novel model of *Fa* transfer dynamics from coronary plasma through the endothelial cells and interstitium to the cardiomyocyte, applying standard physicochemical principles on diffusion and on the chemical equilibrium of *Fa* binding to carrier proteins *Cp*, like albumin in plasma and interstitium and Fatty Acid-Binding Proteins within endothelium and cardiomyocytes. Applying these principles, the present model strongly suggests that in the heart, binding and release of *Fa* to and from *Cp* in the aqueous border zones on both sides of the cell membranes form the major hindrance to *Fa* transfer. Although often considered, the membrane itself appears not to be a significant hindrance to diffusion of *Fa*. Proteins, residing in the cellular membrane, may facilitate transfer of *Fa* between *Cp* and membrane. The model is suited to simulate multiple tracer dilution experiments performed on isolated rabbit hearts administrating albumin and *Fa* as tracer substances into the coronary arterial perfusion line. Using parameter values on myocardial ultrastructure and physicochemical properties of *Fa* and *Cp* as reported in literature, simulated washout curves appear to be similar to the experimentally determined ones. We conclude therefore that the model is realistic and, hence, can be considered as a useful tool to better understand *Fa* transfer by evaluation of experimentally determined tracer washout curves.

## Author Summary

The energy required for pump work of the heart is generated primarily by oxidation of long-chain fatty acids (*Fa*), transferred to the heart by plasma albumin. In coronary capillaries, *Fa* detach from albumin, cross endothelial cells, pericapillary interstitium and cardiomyocyte membrane prior to oxidation. The exact mechanism underlying the transfer process, however, is unknown. We designed a computer model of this *Fa* transfer using parameter values in the physiological range. We postulate that known physical principles of diffusion, *Fa*-protein complex binding, and *Fa* solubility in water and lipid membranes can describe intra-cardiac transfer. Model simulations were compared with multiple indicator dilution experiments, administrating a bolus of labeled *Fa* and albumin into the coronary artery. The resulting dilution time courses of label concentrations in the coronary veins compared favorably with the model simulations. We conclude that the model appears to be realistic, providing a useful tool to study in detail the mechanisms of intra-cardiac *Fa* transfer.

## Introduction

The heart acts as a pump to supply oxygenized blood to all organs in the body. The energy required for cyclic contraction and relaxation of the cardiac muscle cells (cardiomyocytes) is provided by oxidation of blood-borne substrates. Normally, long-chain fatty acids (*Fa*), such as palmitate (C16:0) and oleate (C18:1), constitute the major group of substrates for cardiac energy conversion [1, 2]. *Fa*, supplied to the heart by coronary arterial blood, travel from the capillary lumen through the endothelial and interstitial compartments to the cardiomyocytes. As *Fa* are poorly soluble in water, carrier proteins (*Cp*), like albumin in blood plasma and interstitial fluid and Fatty Acid Binding Proteins (*FABP*) in the cytoplasm of endothelial and cardiac muscle cells, are required to guarantee sufficient supply of *Fa* to the cardiomyocyte interior [3]. Upon arrival in the cytoplasm of the cardiomyocytes, *Fa* are metabolized to acyl-CoA by mitochondrial acyl-CoA synthetase and subsequently oxidized inside the mitochondria for energy conversion, while part of the *Fa* moieties is incorporated in the cardiomyocyte triacylglycerol pool [1, 2].

In the past decades, several crucial steps in *Fa* uptake in the heart have been identified. From the collective data, the following physiological concept emerges. Coronary blood supplies *Fa*, predominantly bound to plasma albumin, to the myocardial capillaries almost exclusively by convection. Due to the high affinity of albumin for *Fa* [4–6] with physiological albumin concentrations of 0.6–0.8 mmol l^-1^, in blood plasma the concentration of total *Fa* (0.2–0.5 mmol l^-1^) exceeds that of free *Fa* by a factor of 10^5^. Since the endothelium, lining the capillary lumen, is virtually impermeable to albumin [3], *Fa* must detach from albumin before passing the endothelial cell membrane. From capillary to cardiomyocyte interior, *Fa* permeate three phospholipid bilayers and three aqueous compartments. The bilayers are the luminal and abluminal endothelial cell membranes and the cardiomyocyte membrane. The aqueous compartments are the endothelial cytoplasm, the pericapillary interstitium, and the cardiomyocyte cytoplasm. Transfer of *Fa* through membranes most likely occurs by diffusion [7]. Because of the very low solubility of *Fa* in water [8], transfer of *Fa* through the aqueous compartments occurs by carrier-mediated diffusion, implying temporary binding of *Fa* to a compartment-specific diffusible carrier protein *Cp* to form the complex *CpFa* [9]. Another more well-known example of carrier-mediated diffusion is that of oxygen transport in blood, facilitated by hemoglobin. Within the capillary compartment and interstitium, albumin plays the role of *Cp*. Within the cytoplasm of the endothelial and cardiac muscle cells, low-molecular weight *Fa* binding proteins (*FABP*) are thought to facilitate *Fa* diffusion [10, 11]. Despite the importance of unimpeded *Fa* supply to the cardiomyocytes, physicochemical understanding of *Fa* transfer from plasma to the cardiomyocyte interior is still incomplete.

Bassingthwaighte and co-investigators [12, 13] were the first to model the entire compound trajectory from arterial inflow to the site of metabolism in the cardiomyocyte, including the outflow of non-metabolized compound through the veins. In the GENTEX version of this model [14] aqueous solutes permeate the endothelial cell membrane by diffusion, possibly supported by carrier-mediated transport. In this model, however, transport of non-soluble *Fa* through the aqueous compartments was not considered to be a hindrance. To fulfill the physiological needs of intra-myocardial *Fa* transfer, among others, the binding reaction of *Fa* to the *Cp* in intra- and extracellular fluids and the related carrier-mediated diffusion should be considered, but these aspects were not included in the GENTEX model. Musters and colleagues [15] extended the GENTEX-type model to *Fa* transfer across the membrane. Their model, however, only considered *Fa* to cross the cell membrane by a flip-flop mechanism. They concluded that the flip-flop rate had to be very high for adequate cardiac *Fa* uptake. Because of the fact that this rate was substantially higher than experimentally found by Cupp and coworkers [16], Musters and colleagues introduced the involvement of a membrane-protein transporter, ignoring other aspects of importance in this *Fa* transfer, not necessarily requiring transporters. Besides, only the sarcolemma was considered. The shortcomings of Musters’ model were thoughtfully summarized by Kamp and Hamilton [17]. Weisiger and co-investigators [18] designed a mathematical model of *Fa* (oleate) transfer from *Cp* (albumin) containing plasma through a stagnant water layer into a lipid space, representing the phospholipid bilayer of a cellular membrane. Barta and co-investigators [19] described a numerical solution using slightly modified versions of Weisiger’s equations. With this model, they evaluated the dependency of *Fa* transfer through the plasma-membrane boundary on the kinetics of *Fa* binding to *Cp* in capillary plasma near the endothelial membrane. To the best of our knowledge, at present there is no model available that fully describes the transfer of lipophilic substances, such as *Fa*, from artery to cardiomyocyte, incorporating kinetics of *Fa* binding to *Cp* in all aqueous compartments with specific *Fa* and *Cp* concentration profiles in the boundary zones near the membranes and including *Fa* transfer through the phospholipid bilayers to be crossed.

The present study was designed to get better insight into the cardiac uptake, and myocardial transport and storage of *Fa*. We have chosen for a unique quantitative modeling approach, incorporating the several interacting mechanisms based upon standard physical and physicochemical principles. The model includes convection by flowing blood, diffusion through cell membranes and aqueous compartments, and metabolic conversion in the cardiomyocytes. The aqueous compartments include plasma, endothelium, interstitium and the cardiomyocyte. *Fa* are known to traverse aqueous compartments by carrier-mediated diffusion, involving binding of *Fa* to compartment-specific *Cp*, but none of the models presented so far incorporated this essential feature for lipophilic substances. Furthermore, as main hindrances for *Fa* transfer, we consider the aqueous boundary zones on both sides of the membranes, where *Fa* bind to or detach from *Cp*.

When presenting a model of a physiological process, the design of the model should allow for experimental testing. In the present study, we tested the model by comparing simulated and experimentally determined trans-coronary washout curves of radioactively-labeled albumin and *Fa* in a multiple indicator dilution experiment at different albumin and *Fa* concentrations in the coronary perfusate. Such a comparison requires a model design that can handle time dependency of *Fa* concentrations in all compartments.

## Results

### Model of *Fa* transfer from artery to cardiomyocyte

#### Background

**Fig 1:**
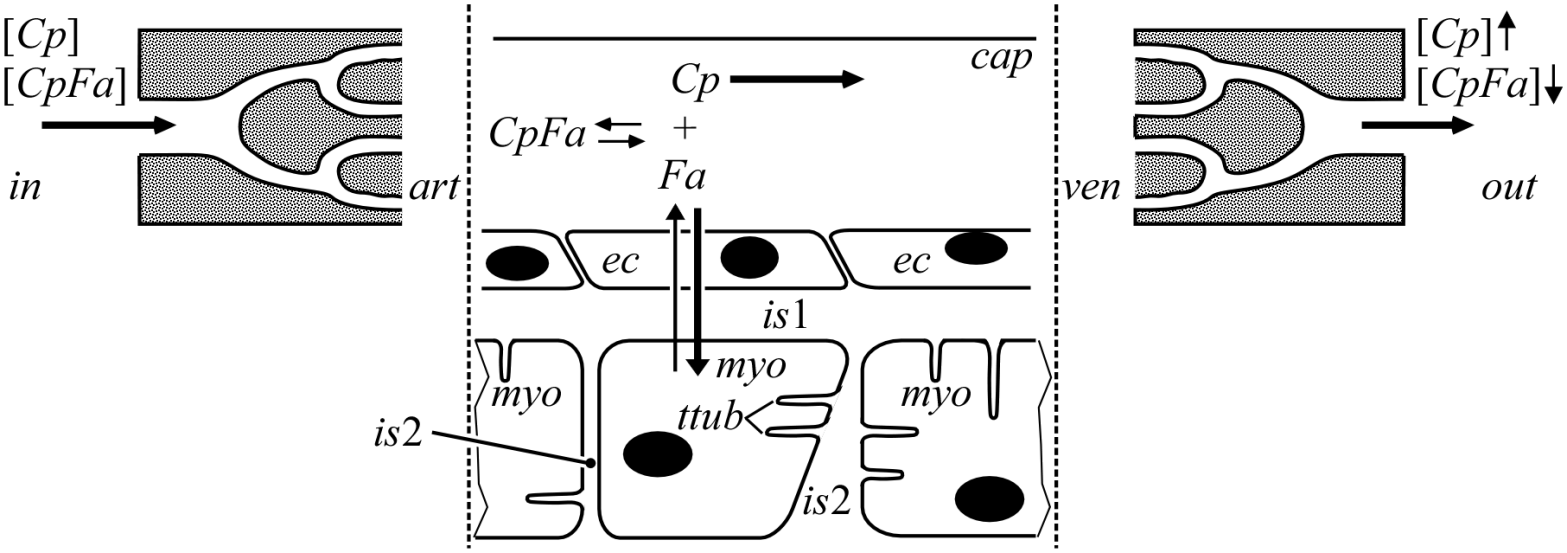
Schematic representation of long-chain fatty acid (*Fa*) transfer in myocardium. *Fa* enter the coronary arteries at location 'in' while predominantly bound to albumin, serving as capillary lumen-specific carrier protein (*Cp*). After entering the capillaries (*cap*) at location '*art*', part of the *CpFa* complexes dissociates, allowing *Fa* transfer to myocardial tissue compartments, *i*.*e*., endothelial cells (*ec*), pericapillary interstitium (*is*1), cardiomyocytes (*myo*), non-pericapillary interstitium (*is*2) and T-tubules (*ttub*). In the latter aqueous compartments, most *Fa* are bound to compartment-specific *Cp*. Non-metabolized *Fa* leave the capillaries mainly as *CpFa* draining into the veins (*ven*). Exchange of *Fa* between plasma and tissue in blood vessels other than the capillaries is neglected.

In Fig 1, a generally accepted concept of cardiac *Fa* uptake is presented based on current physiological and histological knowledge. *Fa*, present in arterial blood, pass coronary arteries (*art*) by convection to enter the capillaries. Nearly all *Fa* are bound to albumin, the *Fa*-carrier protein *Cp* in blood plasma. Capillaries are considered to be the major site of uptake of lipophilic substrates, like *Fa*. Near or at the luminal surface of the capillaries (*cap*), free *Fa*, detached from *Cp*, permeate the *cap*-*ec* membrane, cross the endothelial cell (*ec*) itself, then the *ec-is*1 membrane to enter the pericapillary interstitial compartment (*is*1), and from there across the *is*1-*myo* membrane into the interior of the cardiomyocytes (*myo*), where *Fa* are metabolized. As *Fa* dissolve poorly in water, the aqueous nature of intracellular and interstitial fluid causes the free *Fa* concentration to be so low that diffusion of free *Fa* is quantitatively miniscule. Diffusion of *Fa* through capillary clefts is likewise negligible, whereas diffusion of *CpFa* is also severely hindered [3]. Through all aqueous compartments, *Fa* transfer predominantly occurs by carrier-mediated diffusion, using compartment-specific *Cp*. For the extracellular compartments *cap*, *is*1 and *is*2 (non-pericapillary interstitium) albumin serves as *Cp*, and for the intracellular compartments *ec* and *myo*, cell-specific *FABP* serve as *Cp*. We consider *Fa* to permeate the membranes of the endothelial cells and the cardiomyocyte by diffusion as free molecules [17]. Non-exchanged and non-metabolized *Fa*, present near the outflow end of the capillaries, drain into the veins (*ven*). *Fa*, bound to *Cp*, pass the venous compartment by convection while neglecting exchange with the tissue surrounding the coronary veins.

**Fig 2:**
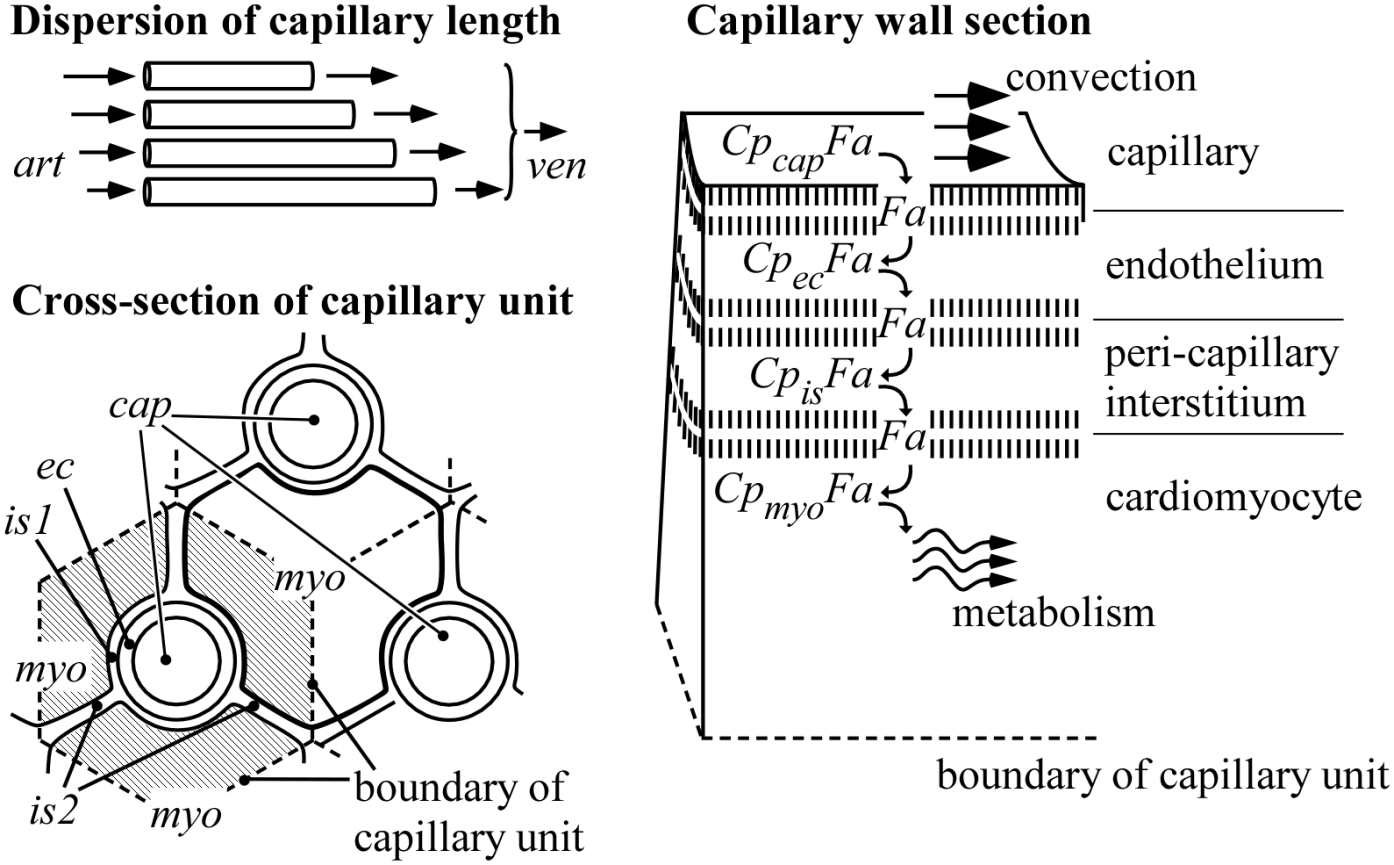
Schematic representation of intramyocardial *Fa* transfer in the capillaries. Top left, "Dispersion of capillary length": Capillaries of different length all drain in the same venous outlet (*ven*). Bottom left, "Cross-section of capillary unit": The region attributed to a capillary (*cap*) defines a capillary unit (shaded). The bold contour marks a cardiomyocyte (*myo*), being surrounded by three capillaries as an example, resulting in subdivision of the cardiomyocyte by the capillary unit boundaries. Abbreviations *ec*, *is*1 and *is*2 indicate endothelium, pericapillary interstitium and non-pericapillary interstitium, respectively. Right, "Capillary wall section". In this section, *Fa* move from capillary lumen through endothelium and pericapillary interstitium to the interior of the cardiomyocyte. Within these aqueous compartments, *Fa* are mainly transferred by carrier-mediated diffusion while bound to a compartment-specific carrier protein *Cp*. Inside the cellular membranes, separating these compartments, *Fa* are dissolved as free molecules and transferred by diffusion. At all 6 aqueous compartment-membrane boundaries *Fa* are exchanged between compartment-specific *Cp* and phospholipids in the membrane. Only inside the cardiomyocyte, a substantial fraction of *Fa* is eventually metabolized.

In modeling trans-coronary *Fa* transfer, we included the following physical and physicochemical principles (Figs 1 and 2):

- In the arteries (*art*) and veins (*ven*), transfer of *Fa* is predominantly blood flow-related convection without uptake in myocardial tissue.
- *Fa* extraction occurs in capillaries only.
- Carrier proteins *Cp*, like albumin and *FABP*, cannot pass cell membranes.
- In capillaries, transfer of capillary *Cp* and *CpFa* from arterial inlet to venous outlet occurs by flow-related convection. Compartments outside the bloodstream are virtually stagnant, having zero fluid flow velocity.
- Variation in flow path length throughout the capillary network is represented by assuming dispersion of capillary length.
- In all aqueous compartments, a compartment-specific *Fa*-carrier protein *Cp* is present. *Fa* pass these compartments mainly by carrier-mediated diffusion, *i*.*e*., complexed with *Cp* to *CpFa*.
- When *Fa* move from an aqueous compartment into a cell membrane, thus passing a water-phospholipid boundary, *Fa* detach from *Cp*, and dissolve into the cell membrane. On the other side of the membrane, this process occurs in reverse direction.
- On their way from capillary to the cardiomyocyte interior, *Fa* encounter 6 water-phospholipid boundaries (two sides per membrane), all modeled by the same mathematical description.
- *Fa* are metabolized in the *myo* compartment only.
- To facilitate modeling of intramyocardial *Fa* transfer, in Fig 2, the concept "capillary unit" is introduced as the capillary *cap* with related endothelium *ec*, pericapillary interstitium *is*1, and capillary related fractions of the cardiomyocyte *myo* and the interstitium *is*2. Since a single cardiomyocyte is surrounded by more than one capillary, boundaries between adjacent capillary units divide the cardiomyocyte.
- The interstitium is subdivided into the compartments *is*1 and *is*2. Although these compartments are connected, their functions are considered very different. *Fa* must cross *is*1 in the transfer from *cap* to *myo*. Compartment *is*2 is surrounded by *myo*, and is less relevant for transfer of *Fa* from the endothelium to the cardiomyocytes because the total surface area of the entrance from the *is*1 to the *is*2 compartment is only about 2% of the surface of the sarcolemma facing *is*1. Moreover, the diffusion distance for *Fa* considerably increases on their way from endothelium to the cardiomyocytes, when transferring *is*2 in lateral direction. However, like the *myo* compartment, *is*2 may store *Fa* temporarily because of its size and relatively high albumin content. In this concept, storage is meant as temporary storage of *Fa* without chemical conversion into other compounds, such as triacylglycerol or phospholipids.
- *Fa* can be stored in significant amounts in aqueous compartments solely by binding to *Cp* and in cell membranes by dissolution as free molecule in the phospholipid bilayer.

These principles are briefly elucidated in the following subsections. The mathematical model was designed to handle time-dependent concentrations of albumin and *Fa*, allowing for evaluation of the model by multiple indicator dilution experiments [20] as described in the section "Testing the model by multiple indicator dilution experiments". In such an experiment, during one or a few seconds a mixture of radioactively labeled albumin and *Fa* (palmitate) was inserted into the perfusion fluid entering the coronary artery of an isolated rabbit heart. The concentrations of the radioactive labels were measured in the coronary venous outlet as a function of time. Analysis of these concentration washout curves provides information about the transport properties of the labeled compounds in the coronary system.

The subsection "Arterio-venous transfer of compounds" deals with smearing out of concentrations in time after injection of labeled indicator compounds at the coronary artery entrance. Subsection "Transfer from capillary lumen to cardiomyocyte" deals with the implementation of *Fa* transfer from capillary through endothelium and pericapillary interstitium to the interior of the cardiomyocyte. The aqueous compartments are separated by cell membranes. *Fa* pass these membranes as free molecules. In subsection "Diffusion of *Fa* through a water-phospholipid boundary", we quantified *Fa* permeability of the boundary zone, being the aqueous layers near the cellular membranes, ascribing a major role to dissociation of the *CpFa* complex in that zone. Subsection "Diffusion transfer of *Fa* through aqueous compartments" describes the summed effect of free and carrier-mediated diffusion of *Fa* through an aqueous compartment. Subsection "Capillary length dispersion" shows how we incorporated this dispersion in the model. In subsection "*Fa* storage capacity in tissue" the ability to store significant amounts of *Fa* is estimated for compartments and, although rarely considered relevant, also for cell membranes. In the ‘Method’ section, the topics discussed in the subsections are elaborated on in more detail, when necessary.

#### Arterio-venous transfer of compounds

Using the schematic representation as described in Fig 1, compound concentration at the coronary entrance [*Comp*]_*in*_ is assumed to be a function of time *t*. In analyzing compound transfer from entrance to the venous outlet *out*, we distinguish compartments formed by the arteries (*art*), capillaries (*cap*) and veins (*ven*). Since blood flow velocity, path length and compound extraction vary within the myocardium, compound concentration is smeared out in time when passing the various compartments.

We described this phenomenon quantitatively as follows. The time-dependent *Comp* concentration at each compartment outlet is found by convolution of the *Comp* concentration at the inlet with an impulse response function *H*(*t*), being defined as the response of outlet concentration to an infinitesimally short concentration peak at the inlet. Referring to Fig 1, for the compartments *art*, *cap* and *ven*, the inlet concentrations are [*Comp*]_*in*_(*t*), [*Comp*]_*art*_(*t*) and [*Comp*]_*ven*_(*t*) and the outlet concentrations are [*Comp*]_*art*_(*t*), [*Comp*]_*ven*_(*t*) and [*Comp*]_*out*_(*t*), respectively. Arterial, capillary and venous impulse response functions are *H*_*art*_(*t*), *H*_*cap*_(*t*) and *H*_*ven*_(*t*), respectively. If we assume that the system behaves linearly, which is the case in the tracer dilution experiments (see below), it follows that

$$\begin{array}{lllll} {\left[Comp\right]}_{art}\left(t\right) & = & {\left[Comp\right]}_{in}\left(t\right) & * & H_{art}\left(t\right)\\ {\left[Comp\right]}_{ven}\left(t\right) & = & {\left[Comp\right]}_{art}\left(t\right) & * & H_{cap}\left(t\right)\\ {\left[Comp\right]}_{out}\left(t\right) & = & {\left[Comp\right]}_{ven}\left(t\right) & * & H_{ven}\left(t\right) \end{array}$$ (1)

The * symbol in Eq (1) indicates mathematical convolution. Elimination of [*Comp*]_*art*_ and [*Comp*]_*ven*_ from Eq (1) by substitution and using the fact that the sequence of convolutions can be transposed without affecting the final result, it follows:

$${\left[Comp\right]}_{out}\left(t\right)={\left[Comp\right]}_{dec}\left(t\right)*H_{cap}\left(t\right)$$

with

$${\left[Comp\right]}_{dec}\left(t\right)={\left[Comp\right]}_{in}\left(t\right)*H_{art}\left(t\right)*H_{ven}\left(t\right)$$ (2)

Compound concentration [*Comp*]_*dec*_(*t*) represents a single function of time, composed of a combination of unknown functions, depending on the coronary flow level and the dimensions and branching patterns of the coronary vascular tree. Since the compound is assumed to stay within arteries and veins and the different compounds are injected together in one single dose, the function [*Comp*]_*dec*_(*t*) is the same for all compounds.

#### Transfer from capillary lumen to cardiomyocyte

In this section, we consider *Fa* transfer from capillary lumen to the cardiomyocyte interior in a group of capillary units assuming dispersion of capillary length (Fig 2). Within aqueous compartments, most *Fa* are bound to carrier protein *Cp* to form complex *CpFa*. A tiny fraction of *Fa* is freely dissolved in water with concentration [*Fa*]. In the state of equilibrium, the concentrations [*Fa*], [*Cp*] and [*CpFa*] are related by the equilibrium constant *K*_*CpFa*_:

$$\left[Fa\right]\;\left[Cp\right]=K_{CpFa}\left[CpFa\right]$$ (3)

Total capillary area *A*_*cap*_(*z*) is defined as the summed cross-sectional area of all capillaries with a length equal to or longer than *z*, representing the axial distance from the capillary entrance. All capillaries are assumed to have the same diameter. So, *A*_*cap*_ is proportional to the number of capillaries included. With increasing *z*, we lose capillaries shorter than *z*. As a consequence, the total cross sectional area *A*_*cap*_ diminishes with increasing value of *z*. Because flow velocity is lower in longer capillaries, mean capillary flow velocity *v*(*z*) also decreases with increasing *z*. Mean total capillary *Fa* concentration [*Fa*_*tot*_]_*cap*_, being the sum of concentrations [*Fa*] and [*CpFa*], and total *Fa* transfer *g*_*cap*,*ec*_ from capillary into the endothelium are both a function of *z* and time *t*. Using conservation of *Fa* mass, it holds:

$$A_{cap}\left(z\right)\;\left(\frac{\partial {\left[Fa_{tot}\right]}_{cap}\left(z,t\right)}{\partial t}+v\left(z\right)\frac{\partial {\left[Fa_{tot}\right]}_{cap}\left(z,t\right)}{\partial z}\right)+g_{cap,ec}\left(z,t\right)=0$$ (4)

Symbols *A* and *g* indicate cross-sectional area [m^2^] and compound transfer [mol m^-1^s^-1^], respectively. Since in the aqueous compartments *ec*, *is*1 and *myo* convection is virtually absent, *v*(*z*) = 0 in these compartments. Compartments *ec* and *is*1 both are sandwiched between two cell membranes (Fig 2), resulting in two *g*(*z*,*t*)-terms. In compartment *myo*, a substantial part of the *Fa* is metabolized, as expressed by function *g*_*met*_. In summary, for compartments *ec*, *is*1 and *myo* we applied:

$$\begin{array}{l} A_{ec}\left(z\right)\frac{\partial {\left[Fa_{tot}\right]}_{ec}\left(z,t\right)}{\partial t}-g_{cap,ec}\left(z,t\right)+g_{ec,is1}\left(z,t\right)=0\\ A_{is1}\left(z\right)\frac{\partial {\left[Fa_{tot}\right]}_{is1}\left(z,t\right)}{\partial t}-g_{ec,is1}\left(z,t\right)+g_{is1,myo}\left(z,t\right)=0\\ A_{myo}\left(z\right)\frac{\partial {\left[Fa_{tot}\right]}_{myo}\left(z,t\right)}{\partial t}-g_{is1,myo}\left(z,t\right)+g_{met}\left(z,t\right)=0 \end{array}$$ (5)

with

$$g_{met}\left(z,t\right)=R_{met}A_{myo}\left(z\right)\;{\left[Fa\right]}_{myo}\left(t\right)$$

Constant *R*_*met*_ represents metabolic rate. Since the cardiomyocyte is surrounded by several capillaries (Fig 2) with possibly different flow directions and different distances from the inflow location, we cannot attribute a meaningful *z-*coordinate to the *myo* compartment. Therefore, in Eq (5), for the *myo*-compartment, we have used a general average of [*Fa*] independent of *z*. Furthermore, because *Fa* energy metabolism in cardiomyocytes largely exceeds that in non-myocytal cells [21, 22], conversion of *Fa* in all other compartments considered was neglected.

#### Diffusion of *Fa* through a water-phospholipid boundary

Transfer of *Fa* by diffusion from an aqueous compartment through the phospholipid membrane (e.g., *g*_*cap*,*ec*_(*z*,*t*) in Eq 4) to the next aqueous compartment requires *Fa* detachment from the *CpFa* complex proximally of the membrane, diffusion through the membrane as free molecules and re-attachment to the compartment-specific *Cp* distally to the membrane. Because *Fa* are metabolized in the cardiomyocyte, average *Fa* transfer is unidirectional. As a consequence, the physicochemical reactions related to attachment and detachment of *Fa* from *Cp* in the boundary zones of the compartments have a unidirectional average, implying that these reactions are not in a state of equilibrium. So, transfer of *Fa* across a membrane requires thermodynamic energy generated by the concentration gradient. Therefore, to quantify *Fa* transfer across a membrane, we may represent the processes in the boundary zones near a membrane by a virtual diffusion resistance, to be quantified by a value for permeability. Total permeability of a membrane separating two aqueous compartments is a composite of the permeabilities of the proximal water-membrane boundary, of the membrane itself, and of the distal water-membrane boundary.

Within a compartment, the concentration drop of free *Fa* acts as the driving force for *Fa* transfer by free *Fa* diffusion as well as by carrier-mediated *Fa* diffusion. Besides, the same concentration drop also acts as driving force for trans-membrane *Fa* transfer. So, free *Fa* concentration in water is considered as the concentration potential for *Fa* diffusion. Thus, total *Fa* flux, expressed in [mol m^-2^s^-1^], equals free *Fa* concentration drop [mol m^-3^] times permeability [m s^-1^]. The latter permeability depends on concentration and *Fa*-binding properties of the *Cp* involved and on *Fa*-affinity of the membrane.

**Fig 3:**
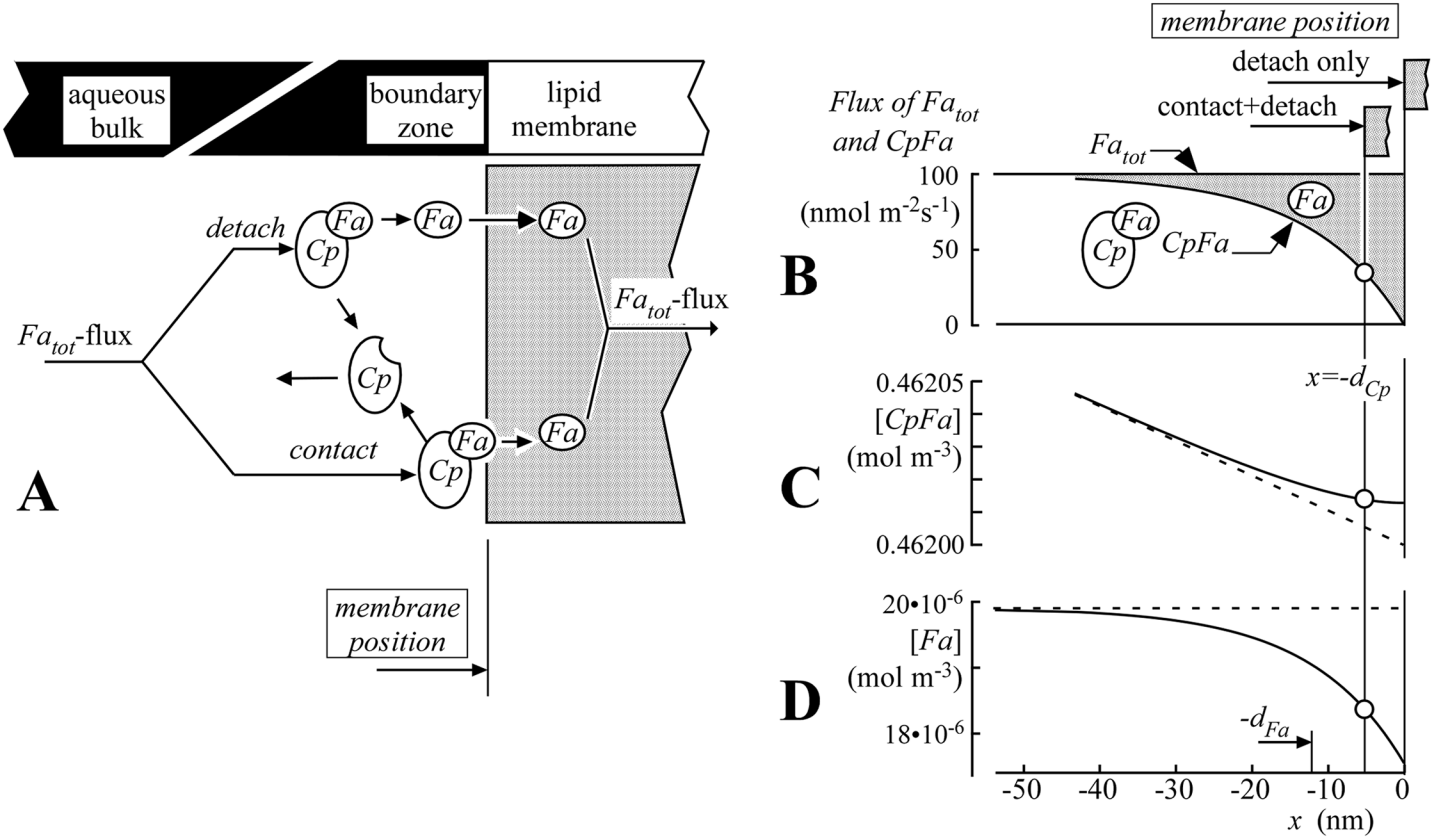
Permeability of water-phospholipid membrane boundary for *Fa*. In panel A, transfer mechanisms 'detach' and 'contact' indicate *Fa* detachment from *CpFa* in the aqueous boundary zone followed by diffusion of free *Fa* towards the cellular membrane ('detach pathway') and *Fa* delivery from *CpFa* to the phospholipid bilayer of the cellular membrane by direct contact ('contact pathway'), respectively. Panels B-D refer to a physiologically realistic situation in the boundary zone between the capillary plasma and endothelial cell membrane with plasma albumin concentration 0.66 mmol l^-1^, and total *Fa* concentration 0.60 mmol l^-1^. Solid lines in graphs B, C and D represent *Fa*-flux (Eqs 17, 19 and 20) and concentrations [*CpFa*] (Eq 23) and [*Fa*] (Eq 28), respectively, as a function of its position relative to the boundary x = 0. If only the detach pathway is active, the membrane surface is positioned at x = 0. In the boundary zone the fraction of total *Fa*-flux, occurring by diffusion of free *Fa* (shaded fraction in B) increases exponentially with distance constant *d*_*Fa*_ to 100% at x = 0 at the cost of the fraction of carrier-mediated diffusion. Relative variations in [*CpFa*] are little, while those for [*Fa*] are considerable (C and D, respectively). Dashed lines indicate linear extrapolations of concentration profiles in the aqueous bulk. If both the detach and contact pathways are active, the membrane surface is positioned at x = −*d*_*Cp*_, representing the membrane reaction rate parameter. Then, at the membrane surface, part of the total *Fa* flux occurs by *CpFa* flux towards the membrane, followed by delivery of *Fa* into the membrane through direct contact between *CpFa* and membrane. While keeping *Fa*_*tot*_ flux constant, an increase of *d*_*Cp*_ induces lowering of the free *Fa* concentration drop relative to the bulk concentration (D), implying an increase of boundary zone permeability *P*_*b*_, being defined as flux divided by concentration drop. It is of note that with inversion of *Fa* flux (“back flux”) all processes and concentration gradients are reversed too, in other words the model is symmetric.

In our derivation, permeability *P*_*b*_ (Eq 6) is associated with the boundary between aqueous fluid and membrane phospholipids. If *Fa* flux is directed towards the membrane, in the fluid zone near the boundary ('boundary zone' in Fig 3A) *Fa* detach from the *CpFa* complex and diffuse freely through the water before entering the membrane (the so-called 'detach' pathway'). Following an alternative pathway (the so-called 'contact pathway'), the *CpFa* complex delivers *Fa* to the membrane by direct physical contact. Total *Fa* flux is the sum of free *Fa* flux and *CpFa* flux. As derived in detail in the method section, when approaching the boundary from the aqueous side, the fraction of free *Fa* flux increases exponentially at the cost of the fraction of *CpFa* flux, the latter representing carrier-mediated diffusion (Fig 3B). Furthermore, in this so called boundary zone, the concentration of free [*Fa*] drops considerably, whereas the relative decrease in concentration [*CpFa*] is practically negligible. *Fa* transfer due to direct contact between *CpFa* and the phospholipid membrane implies that *CpFa* are transferred to the boundary, while an equal amount of unbound *Cp* is transferred away from the membrane. The rate of the *CpFa*-phospholipid contact reaction is expressed by the membrane reaction rate parameter *d*_*Cp*_, having the physical dimension of length and being defined more precisely in relation to Eq (24). The value of *d*_*Cp*_ depends on the type of *Cp* and the properties of the membrane. In the method section, it is derived that in the boundary zone, free *Fa* concentration decays exponentially with the distance from the fluid-lipid boundary with decay distance constant *d*_*Fa*_. Boundary permeability *P*_*b*_ and exponential decay distance *d*_*Fa*_ depend on diffusion coefficient *D*_*Fa*_ of free *Fa* in plasma, equilibrium constant *K*_*CpFa*_, dissociation time constant *τ*_*CpFa*_, unbound *Cp* concentration [*Cp*] and membrane reaction rate parameter *d*_*Cp*_:

$$P_{b}\approx \frac{D_{Fa}}{d_{Fa}}\left(1+\frac{d_{Cp}}{d_{Fa}}\right)$$

with

$$d_{Fa}=\sqrt{\frac{D_{Fa}K_{CpFa}\;\tau_{CpFa}}{\left[Cp\right]}}$$ (6)

Diffusion coefficient *D*_*Fa*_ expresses the importance of free *Fa* diffusion in the aqueous boundary zone. For crossing a membrane, *Fa* must pass two water-phospholipid boundaries. Applying Eq (6) for each boundary, trans-membrane *Fa* transfer *g*_c1,c2_(*z*,*t*) from compartment *c*1 to compartment *c*2 depends on the concentration drop of free *Fa* by:

$$g_{c1,c2}\left(z,t\right)=\frac{1}{1/P_{b,c1}+\;1/P_{b,c2}+\;1/P_{mem}}\frac{dS_{mem}\left(z\right)}{dz}\;\left({\left[Fa\right]}_{c1}\left(z,t\right)-{\left[Fa\right]}_{c2}\left(z,t\right)\right)$$ (7)

Symbol *S*_*mem*_(*z*) represents the surface area of the membrane considered, summed over all capillaries from the capillary entrance (*z* = 0) until the given *z*-value. Derivative d*S*_*mem*_/d*z* represents the change in membrane surface area with the increase of *z*, which is equivalent to the summed membrane circumference (See also the section 'Capillary length dispersion' below). Like *A*_*cap*_, the value of d*S*_*mem*_/d*z* decreases with increasing value of *z* due to the decreasing number of capillaries included. Symbol *P*_*mem*_ represents permeability of the phospholipid membrane itself. We applied the rule that overall permeability of individual permeabilities in series equals the reciprocal of the sum of reciprocals of these permeabilities. In the model Eq (7) has been applied to all three membranes, as exemplified in Fig 2. The fact that permeability is independent of the direction of transfer, implies that the model handles *Fa* back flux.

#### Diffusion of *Fa* through aqueous compartments

Since the concentration of free *Fa* in water is very low, transfer of free *Fa* by diffusion is insufficient to fulfill the physiological needs of the cardiomyocytes. Therefore, *Fa* transfer must be facilitated by compartment-specific *Cp* in all aqueous compartments (*cap*, *ec*, *is*1 and *myo*). In the methods section it is derived that permeability *P*_*aqu*_ of an aqueous layer with thickness *h*_*aqu*_ depends on diffusion coefficients *D*_*Cp*_, *D*_*Fa*_, equilibrium constant *K*_*CpFa*_, concentration [*Cp*] of free *Cp* and concentration [*Cp*_*tot*_] of total *Cp* by:

$$P_{aqu}=\frac{1}{h_{aqu}}\left(D_{Fa}+D_{Cp}\frac{{\left[Cp\right]}^{2}}{\;K_{CpFa}\;\left[Cp_{tot}\right]}\right)$$ (8)

Since we consider free *Fa* concentration as the potential for diffusive transfer of *Fa*, we calculated total *Fa* transfer through an aqueous compartment as free *Fa* concentration drop over the aqueous layer, multiplied by *P*_*aqu*_.

#### Capillary length dispersion

The length of the flow path through the capillary network varies. Capillary diameter and arterio-venous pressure drop are considered to be the same everywhere in the myocardial tissue. So, for the longer pathways flow velocity will be lower than in the shorter ones due to a smaller pressure gradient in the former, thus introducing flow heterogeneity. We modeled the capillary network as a set of parallel capillary units with the same diameter, but with different lengths (Fig 2). We assumed the logarithm of capillary length to be normally distributed with dispersion parameter *σ*_*cap*_. In the equations below, mean capillary length *z*_*0*_ is used as reference length. In solving the system of differential equations in Eqs (4, 5 and 7), expressions are needed for total capillary cross-sectional area *A*_*cap*_(*z*), total capillary circumference *dS*_*cap*_(*z*)/d*z*, and mean capillary blood flow velocity *v*(*z*). We also assumed that in individual capillaries, flow velocity is inversely proportional to capillary length, *z*. Both *A*_*cap*_(*z*) and total blood flow *q*(*z*) diminish with an increase of *z*, because of a decreasing number of capillaries included. In the method subsection 'Capillary length dispersion' we derived:

$$A_{cap}\left(z\right)=\frac{1}{2}\left(1-Erf\left(\frac{\sigma_{cap}}{4}+\;\frac{\text{ln}\left(z/z_{0}\right)}{\sigma_{cap}}\right)\right)\;\frac{V_{cap}}{z_{0}}$$

with

$$Erf\left(x\right)=\frac{2}{\sqrt{\pi }}\int_{0}^{x}\text{exp}\left(-t^{2}\right)dt$$ (9)

$$q_{cap}\left(z\right)=\frac{1}{2}\left(1-Erf\left(\frac{3\sigma_{cap}}{4}+\;\frac{\text{ln}\left(z/z_{0}\right)}{\sigma_{cap}}\right)\right)\;q_{tot}$$ (10)

$$\frac{dS_{cap}\left(z\right)}{dz}=A_{cap}\left(z\right)\frac{S_{cap,tot}}{V_{cap}}$$ (11)

$$v\left(z\right)={q_{cap}\left(z\right)/A}_{cap}\left(z\right)$$ (12)

Symbol *z*_0_ represents mean capillary length. Symbol *Erf* (*x*) indicates the Error function, representing a smooth step from -1 to +1 around x = 0. Scaling constants *V*_*cap*_, *S*_*cap*,*tot*_, and *q*_*tot*_ indicate total capillary volume, total capillary wall surface area and total capillary blood flow, respectively. In Eq (5) the size of all other compartments (expressed by *A*_*ec*_, *A*_is1_, *A*_*myo*_) and membrane areas within the capillary unit are scaled to *A*_*cap*_(*z*) by a factor equal to the volume fraction ratio of the compartment considered and the capillary compartment. The volume fractions are obtained from ultrastructural findings in the rabbit left ventricular myocardium (Table 1, [23]).

**Table 1:** Ultrastructural data of myocardial tissue.

**Compartment**	Subscript	Fractional	Permeability
		Volume	Thickness
		*V*_*c*_/*V*_*all*_	*h*_*diff*_
		%	nm
Capillaries	*cap*	9.4	
Endothelium	*ec*	1.8	187
Pericapillary interstitium	*is*1	1.9	160
Non-pericapillary interstitium	*is*2	6.0	
Cardiomyocyte compartment	*myo*	73.1	
T-tubules	*ttub*	1.0	
Large blood vessels	*lv*	5.9	
Interstitial cells	*isc*	1.0	
**Surface**	Surface Area		
	*S*/*V*_*all*_		
	m^2^/m^3^		
*cap-ec* membrane	75,200		
*ec-is*1 membrane	82,200		
*is*1*-myo* membrane	89,100		
*is*2 mid-compartment *^)^	94,000		

Data presented in this Table are the mean values obtained from [23].

*^)^ Note that on both sides the *is*2 compartment is bordered by membranes of adjacent cardiomyocytes.

#### *Fa* storage capacity in tissue

In the transfer of *Fa* from capillary to cardiomyocyte and *vice versa*, temporary storage of *Fa* is an important determinant of temporal behavior of *Fa* concentration. By this type of storage, physicochemical binding of *Fa* to a cognate protein or free *Fa* dissolved in lipids are meant; not to be confused with storage by enzymatic change of molecular structure, *i*.*e*., incorporation into the esterified lipid pool. Since solubility of *Fa* in water is very low, *Fa* are stored in physiologically significant amounts by binding to a carrier protein *Cp* or by dissolution in cellular phospholipid membranes. For the aqueous compartments, we used Eq (3) to relate free to total *Fa* concentrations. The total amount of *Fa* stored in a compartment *c* equals total *Fa* concentration multiplied by compartment volume. We quantified storage capacity for *Fa* as the dimensionless ratio *V*_*Fa*,*c*_/*V*_*all*_, representing the total amount of *Fa* that can be stored in compartment volume *V*_*c*_ divided by the amount of *Fa* that can be stored as free *Fa* in the corresponding volume *V*_*all*_:

$$\frac{V_{Fa,c}}{V_{all}}=\frac{\left[Fa_{tot}\right]\;V_{c}}{\left[Fa\right]\;V_{all}}\approx \frac{\left[Cp\right]\;V_{c}}{K_{CpFa}\;V_{all}}$$ (13)

Volume *V*_*all*_ represents the volume of the tissue and blood compartments together. Although the cell membrane is thin (*i*.*e*., the thickness of a phospholipid bilayer, 5 nm, Table 2), implying membrane volume *V*_*mem*_ to be small, *Fa* storage capacity may be physiologically significant since the concentration ratio [*Fa*]_*mem*_/[*Fa*]_*aqu*_, representing the partition factor of the membrane material relative to the plasma, is found to be very high, for palmitic acid for example 8∙10^5^ [8].

**Table 2:** Physicochemical properties of myocardial constituents.

Variable	Symbol	SI-unit	Value	Reference / Remark
***General***				
Diameter capillary	*d*_*cap*_	μm	5.2	[23]
Thickness cell membrane	*h*_*mem*_	nm	5.0	^1)^
Partition coefficient phospholipid/water ^4)^	*C*_*lipid*_	-	8∙10^5^	[8]
Diffusion coefficient *Fa* ^4)^	*D*_*Fa*_	m^2^s^-1^	4.8∙10^−10^	[11, 24]
Number of *Fa* binding sites on albumin	*n*_*Alb*_	-	3	[25, 26]
Mol weight palmitic acid	*M*_*Fa*_	g mol^-1^	256	
***Compartment cap*, *is*1**				
Diffusion coefficient *Cp* (albumin)	*D*_*Cp*,*cap*_	m^2^s^-1^	9.35∙10^−11^	[24]
Equilibrium constant *CpFa* ^4^ ^,^ ^5)^	*K*_*CpFa*,*cap*_	mol m^-3^	9∙10^−6^	[5, 26]
Dissociation time constant *CpFa* ^4)^	*τ*_*CpFa*,*cap*_	s	0.08–0.14	[10, 27]
Mol weight *Cp* (albumin)	*M*_*Cp*,*cap*_	-	67000	[28]
Membrane reaction rate parameter *CpFa*	*d*_*Cp*,*cap*_	nm	5	^2)^
Concentration *Cp* in *cap*	[*Cp*]_*cap*_	mol m^-3^	2.1	*n*_*Alb*_ x [*Alb*] ^3)^
Concentration *Cp* in *is*1	[*Cp*]_is1_	mol m^-3^	86% of *cap*	[29]
***Compartment myo***				
Diffusion coefficient *Cp*	*D*_*Cp*,*myo*_	m^2^s^-1^	1.87∙10^−10^	2×*D*_*Cp*,*cap*_
Equilibrium constant *CpFa*	*K*_*CpFa*,*myo*_	mol m^-3^	9∙10^−6^	[10]
Dissociation time constant *CpFa*	*τ*_*CpFa*,*myo*_	s	0.08–0.14	similar to *cap*
Mol weight *Cp*	*M*_*Cp*,*myo*_	-	15000	[10]
Concentration *Cp*	[*Cp*]_*myo*_	mol m^-3^	0.170	[11]
***Compartment ec***				
Diffusion coefficient *Cp*	*D*_*Cp*,*ec*_	m^2^s^-1^	1.87∙10^−10^	similar to *myo*
Equilibrium constant *CpFa*	*K*_*CpFa*,*ec*_	mol m^-3^	9∙10^−6^	similar to *myo*
Dissociation time constant *CpFa*	*τ*_*CpFa*,*ec*_	s	0.08–0.14	similar to *myo*
Mol weight *Cp*	*M*_*Cp*,*ec*_	-	15000	similar to *myo*
Concentration *Cp*	[*Cp*]_*ec*_	mol m^-3^	0.007	≤4% *myo* [30]

^1)^ https://en.wikipedia.org/wiki/Lipid_bilayer (Nov 26, 2015)

^2)^ Average radius of *Alb*, open to discussion, see text

^3)^ This value refers to the physiological concentration of albumin in blood plasma. Under experimental conditions, this value may vary.

^4)^ Palmitic acid

^5)^ Restricted to high-affinity binding sites

### Testing of the model by multiple indicator dilution experiments

#### Multiple indicator dilution experiments

The model describes how an indicator, supplied at the entrance, is distributed as a function of time over the various compartments within the myocardium. To test our model we have used the experimental results of three multiple indicator dilution experiments. In such experiment, in a few seconds a mixture of several indicator compounds enters the coronary arteries of an isolated saline perfused rabbit heart (Fig 1, point '*in*'). The time course of indicator concentrations at the venous outlet (Fig 1, point '*out*') renders important information about the convection-diffusion properties of the system in between.

Experiments were performed on two isolated rabbit hearts. The coronary arteries were perfused with oxygenated Krebs-Henseleit-bicarbonate solution with albumin (*Alb*) and *Fa* at various concentrations of *Alb* and *Fa*. In one experiment, the heart was perfused with *Alb* and *Fa* concentrations of 0.11 and 0.10 mmol l^-1^, respectively. In this experiment coronary perfusate flow was 0.035 ml s^-1^ per ml of myocardium, where the myocardium is considered to include all tissue and fluid compartments. In another experiment the heart was perfused with *Alb* and *Fa* concentrations of 0.0147 and 0.0133 mmol l^-1^, respectively, and thereafter of 0.44 and 0.40 mmol l^-1^, respectively. In the latter two experiments coronary perfusate flow was 0.061 ml s^-1^ per ml of total myocardium.

For *Fa*, palmitic acid was used as a typical fatty acid readily consumed by the heart *in vivo* [1]. During about one second after *t* = 0, part of the arterial *Alb* and *Fa* was replaced by radioactively labeled *Alb* ^*L*^ and *Fa* ^*L*^ keeping the total chemical composition exactly the same. We collected the coronary venous effluent in a sampling system at a rate of 1.0 s^-1^ for the first 30 samples, and at a rate of 0.25 s^-1^ for the next 30 samples. In the collected samples, measured radioactivity of the *Alb* ^*L*^ was converted to a normalized concentration of *Alb* ^*L*^, so that the time integral was equal to 100%. Using the measured ratio of radioactivity of *Fa* ^*L*^ and *Alb* ^*L*^ in an arterial sample, and assuming that *Alb* passes the coronary circulation without being absorbed, measured radioactivity of the *Fa* ^*L*^ was converted to the normalized concentration of *Fa* ^*L*^. The time integral of normalized concentration *Fa* ^*L*^ was always less than 100% because part of *Fa* is metabolized. Supporting files S1 Data, S1 Read and S1 Model provide detailed information about the model test, as programmed with the software package Mathematica 7 (Wolfram Research).

**Fig 4:**
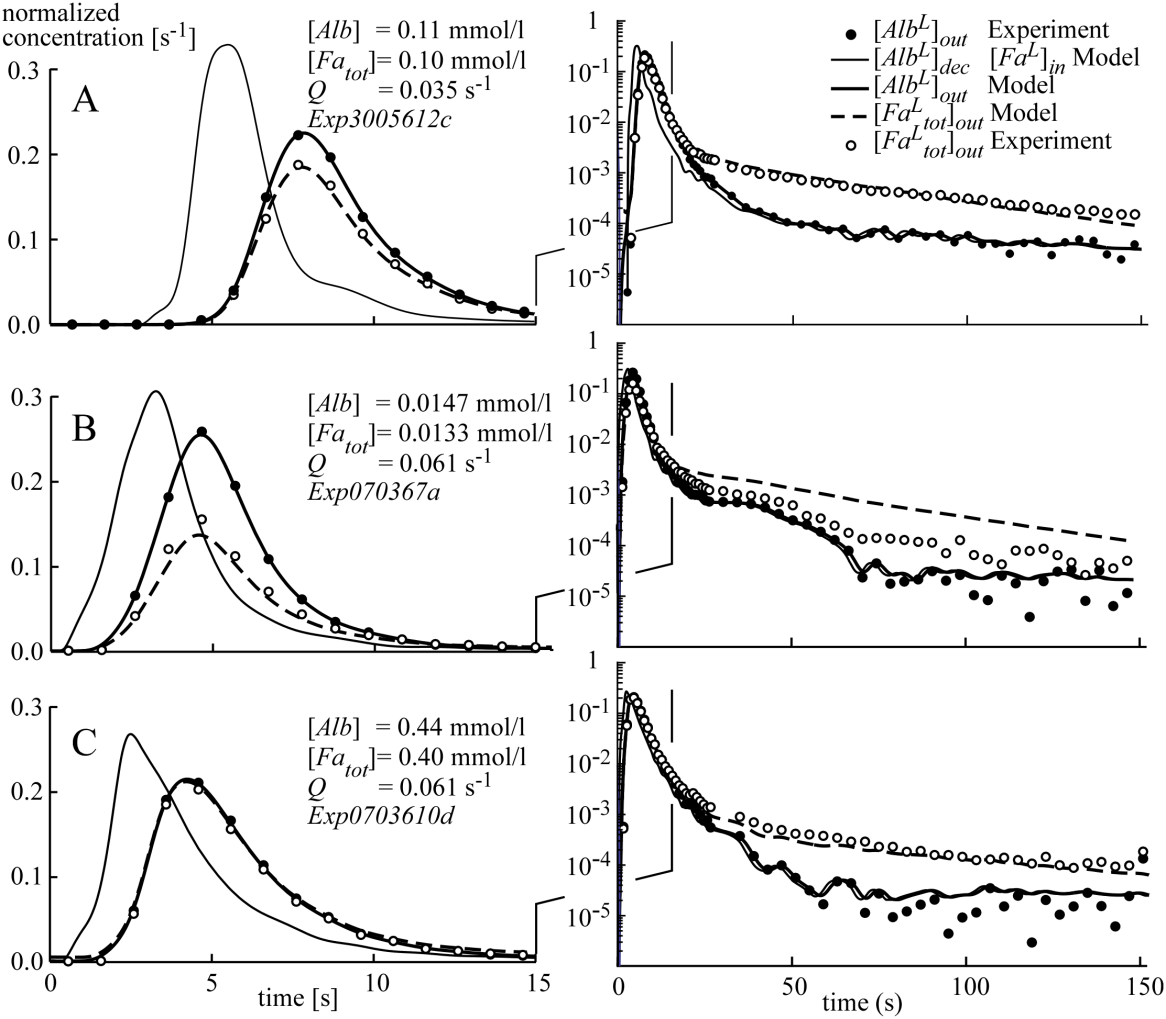
Three examples (A-C) of dilution curves for labeled albumin [*Alb* ^*L*^] and palmitate [*Fa* ^*L*^_*tot*_], on a linear scale to focus on the peak (left panels) and on a logarithmic scale to focus on the tail (right panels). Arterial plasma concentrations [*Alb*]_*in*_ and [*Fa*_*tot*_]_*in*_ and normalized flows Q, expressed in ml s^-1^ per ml of tissue (s^-1^), are indicated in the left panels. Panels B and C refer to the same heart, applying different *Alb* and *Fa* concentrations. From sampled data [*Alb* ^*L*^]_*out*_, capillary inlet concentration [*Alb* ^*L*^]_dec_ was estimated by deconvolution. Using this time course, the model rendered output concentrations [*Alb* ^*L*^]_*out*_ and [*Fa* ^*L*^_*tot*_]_*out*_. For *Alb* ^*L*^, simulation and experiment fit very well. For *Fa* ^*L*^_*tot*_, the fit is good considering the fact that for all three simulations the same set of parameters is used without any experiment-specific fit. Tracer concentration on the y-axis is expressed in [s^-1^] due to normalization by setting the time integral of concentration at the coronary artery entrance equal to 1. *Fa* extraction fraction was found to be 0.12, 0.32 and 0.02 for experiments A, B and C, respectively.

Fig 4 shows the three runs of sampled *Alb* ^*L*^ and *Fa* ^*L*^_*tot*_ output concentrations, indicated by closed and open circles. The first run (Fig 4A) represents an experiment with an intermediate albumin concentration in one heart. The second and third runs (Fig 4B and 4C) are obtained from another heart, one run (4B) with a very low albumin concentration and the other run (4C) with a nearly physiological albumin concentration. The concentration peaks of the curves are shown on a linear scale in the left panels of these figures. For better judgment of the very low concentrations in the tail of the curve (*t*>15s), the same concentration curves are plotted on a logarithmic scale in the right panels of these figures.

Using the model of trans-capillary transfer for *Alb* ^*L*^ assuming zero transfer of *Alb* through the endothelium, we determined the *Alb*-specific impulse response function *H*_*cap*_(*t*) in Eq (1) by solving Eq (4) while replacing *Fa*_*tot*_ by *Alb* ^*L*^ and setting *g*_*cap*,*ec*_(*z*,*t*) = 0. Labeled albumin concentration [*Alb* ^*L*^]_*dec*_(*t*) is found by deconvolution [31] with *H*_*cap*_(*t*) according to Eq (2) using *Alb* ^*L*^ as compound. The result is shown in Fig 4. As a check to the deconvolution procedure, we simulated the concentration [*Alb* ^*L*^]_*out*_(*t*), using calculated [*Alb* ^*L*^]_*dec*_(*t*) as input for the model described by Eq (4). In Fig 4, the thus derived time course [*Alb* ^*L*^]_*out*_(*t*) is found to coincide accurately with the sampled values. Since the indicators *Alb* ^*L*^ and *Fa* ^*L*^ are administered simultaneously, the time courses of normalized indicator concentration [*Alb* ^*L*^]_*dec*_(*t*) and [*Fa* ^*L*^_*tot*_]_*dec*_(*t*) are identical as mentioned in relation to Eq (2). Subsequently, the model of capillary transfer (Eqs 3–12) was applied to *Fa* ^*L*^_*tot*_, resulting in a simulation of labeled *Fa* concentration [*Fa* ^*L*^_*tot*_]_*out*_(*t*). The model was tested by comparing the latter simulated time course with samples of this time course as determined experimentally (Fig 4). In the subsections below we explain in more detail how the data is analyzed.

#### Simulation of steady state carrier protein (*Cp*) concentration

Transfer of *Fa* is quite different from that of *Alb*, since in contrast to *Alb*, *Fa* are able to cross the endothelium, pericapillary interstitium and cardiomyocyte where they are metabolized. Before solving the dynamics of *Fa* ^*L*^_*tot*_ concentration, steady state concentrations of unlabeled free *Cp* are determined for all compartments. Since the concentration of unlabeled free *Cp* is stationary, by application of Eqs (6) and (8) all permeabilities appear stationary. As a consequence, the system of differential equations, determining *Fa* ^*L*^ exchange (Eqs 4 and 5) behaves linearly. A very important consequence of this linearity is that application of the convolutions, as presented in Eqs (1 and 2), is allowed for all time-dependent *Fa* ^*L*^ concentrations in the system. In the capillary plasma and the interstitium, *Alb* serves as *Cp*. Since a single albumin protein has three high affinity binding sites of primary importance (*n*_*Alb*_ = 3, Table 2), the capillary and interstitial binding concentrations of *Cp* are effectively three times higher than the albumin concentration.

For the concentrations of free *Cp* in all compartments, the stationary state is simulated by elimination of time dependency from Eqs (4 and 5). In the multiple indicator experiments, the steady state total concentrations of *Alb* and *Fa* in the perfusion buffer are known. The extraction fraction of *Fa* represents the ratio of missing total *Fa* versus the total amount of *Fa*, entering the capillaries. The non-metabolized fraction of *Fa* ^*L*^, passing the coronary circulation was found by integration of the experimentally observed *Fa* ^*L*^ dilution curve (Fig 4). Metabolic rate constant *R*_*met*_ (Eq 5) was tuned so that *Fa* extraction in the model became equal to the experimentally determined *Fa* extraction, calculated as 100% minus the non-metabolized *Fa* ^*L*^ fraction.

#### Simulation of labeled fatty acid (*Fa* ^*L*^_*tot*_) dilution

As mentioned above, using the *Alb* ^*L*^ dilution curve, *Alb* ^*L*^ concentration at the capillary entrance [*Alb* ^*L*^]_*dec*_(*t*) was estimated as a function of time by deconvolution and normalized so that the time integral became equal to 100%. At this location, the time courses of normalized concentrations [*Fa* ^*L*^_*tot*_]_*dec*_(*t*) and [*Alb* ^*L*^]_*dec*_(*t*) were set identical, because both labeled compounds were mixed before administration at the coronary entrance (‘in’ in Fig 1). In all compartments, we estimated steady state concentrations of free *Cp* using the analysis as discussed above.

In our model, diffusion transfer of total *Fa* ^*L*^_*tot*_ between and within aqueous compartments is driven by differences in concentration of free *Fa* ^*L*^. We calculated permeability of membranes, including effects of the aqueous boundary zones, with Eqs (6 and 7). Trans-aqueous compartment permeability *P*_*aqu*_ was determined by Eq (8). The values of most of the model parameters are known from direct measurements and considered to be accurate. In Table 1 volume fraction of the various compartments, and surface area and thickness of the membranes are obtained from a histological study on the rabbit myocardium [23]. Table 2 shows the values of the parameters as obtained from the referenced literature reports and of a limited number of parameters estimated by us based on reasoning. Some parameter values, however, are subject to discussion. Under physiological circumstances, the *Alb* concentration in compartment *is*1 is normally about 70–86% of that in the capillary lumen [29, 32, 33], considering transudate composition to represent that of interstitial fluid. We used the data of Tschubar *et al* [29], because they experimentally confirmed the ratio of 86% to be valid in a large range of arterial *Alb* concentrations (0.033–0.394 mol m^-3^). Taking the transudate composition of solutes to be representative of that in the interstitial fluid seems to be allowed, because Kroll and colleagues found them to be the same in experiments on isolated guinea pig hearts when local production of these solutes could be neglected [34]. *Alb* has three prominent binding sites for *Fa*, each with a different equilibrium constant for *Fa*-binding [25, 26]. We used the average value of the latter three constants for palmitate. For heart-type *FABP*, identified as the *Cp* in the cardiomyocyte [35], the equilibrium constant has been reported [10]. Assuming similar properties of the *FABP* in endothelial cells and cardiomyocytes, for both cells the same equilibrium constant was used in our model. The dissociation time constant of a *CpFa* complex cannot be determined easily. For *Alb* a value of the dissociation time constant has been reported [10, 27], but it is derived from experiments where membrane-vesicles and fast mixing are involved, possibly limiting the response time of the measurement. Information on the dissociation time constant of *FABP* is lacking. Therefore, we used the corresponding value of *Alb* for *FABP*. For the endothelium, the concentration of *FABP* is not well known, but is reported not to exceed 4% of the *FABP* concentration in the cardiomyocyte [30]. In our first simulation (Fig 4A), the unknown concentration [*Cp*]_*ec*_ has been adjusted for a best fit between model and measurement of the time course [*Fa* ^*L*^_*tot*_]_*out*_(*t*). For the additional two simulations (Fig 4B and 4C), concentration [*Cp*]_*ec*_ was kept at the same value. For all compartments, the diffusion coefficient *D*_*Cp*_ may be overestimated due to the hindering effect of the intra- and extra-cellular structures on protein mobility. Membrane reaction rate parameter *d*_*Cp*_ is not known, but was assumed to be equal to the average radius of *Alb*, being about 5 nm [36, 37].

Fig 4 shows three examples of simulated *Fa* ^*L*^_*tot*_ and *Alb* ^*L*^_*out*_ concentrations as a function of time, obtained from two different hearts. When comparing predicted and measured *Fa* ^*L*^_*tot*_ concentrations, the shape of peak and tail as simulated appear in good agreement with the measurements, considering the fact that for the different curves the same set of model parameters has been used. So, no curve-specific parameter estimation has been applied. The variations between model and experiment in the tail of the experiment shown in Fig 4B are due to uncertainties as a consequence of the very low concentrations we are dealing with in this phase. Comparison of the predicted and measured *Alb* ^*L*^_*out*_ concentrations revealed very good agreement between the shape of the simulated and measured peaks and tails. The latter demonstrates the adequacy of the deconvolution procedure.

#### Sensitivity analysis

To compare model simulations of indicator dilution curves with experimental observations a sensitivity analysis was designed describing how the simulated dilution curves depend on relative variations of the model parameters. We performed the analysis around conditions, most favorable to obtain information on intramyocardial *Fa* transfer properties by analysis of the above mentioned indicator dilution experiments. The *Alb* concentration in the perfusate should not be too high, because then the *Fa* extraction fraction is so low that *Fa* uptake cannot be determined reliably. On the other hand, the *Alb* concentration should not be too low, because then the *Fa* binding capacity is insufficient. A compromise was found by using *Alb* and total *Fa* concentrations of 0.11 and 0.10 mmol l^-1^, respectively, the dilution curves of which are shown in Fig 4A.

Because the model makes use of many parameters, the analysis has been structured so that the sensitivity of most of these parameters can be deduced from the provided sensitivity data. First, we investigated the effect of relative variations in total flow *q*_*tot*_ (Eq 10) and in relative dispersion *σ*_*cap*_ of capillary length (Eq 9), respectively. Furthermore, sensitivity to relative variations in concentration [*Cp*] and dissociation time constant *τ*_*CpFa*_ were calculated for the four compartments *cap*, *ec*, *is*1 and *myo*.

**Fig 5:**
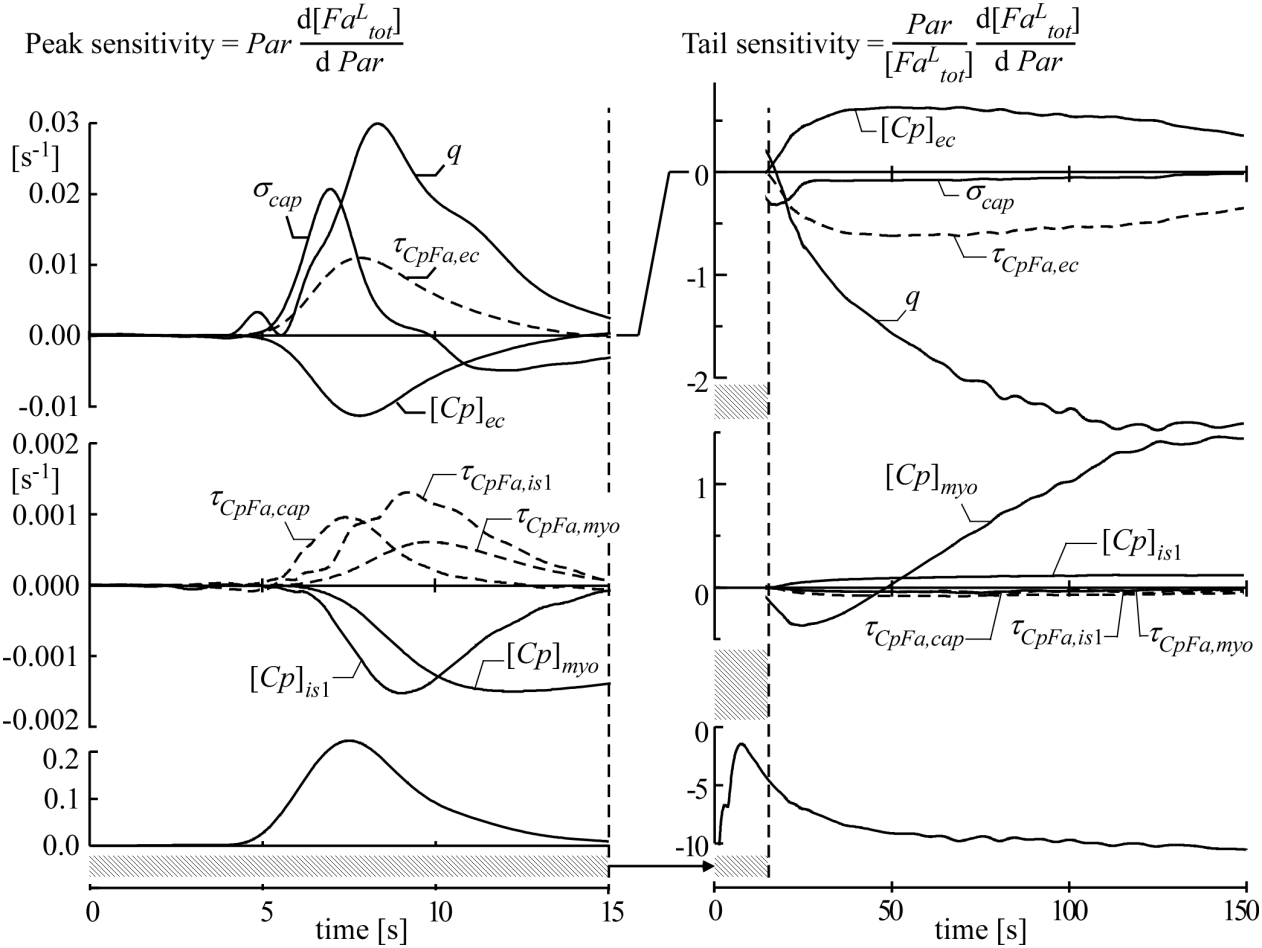
Sensitivity of total labeled *Fa* ^*L*^_*tot*_ concentration in the venous effluent to parameter (*par*) variations. In the upper two left panels, sensitivity *par* ∂[*Fa* ^*L*^_*tot*_]*/*∂*par* is shown for the peak. In the upper two right panels sensitivity (*par/*[*Fa* ^*L*^_*tot*_]) ∂[*Fa* ^*L*^_*tot*_]*/*∂*par* is shown for the tail. The shaded areas indicate the time interval of the peak. Abbreviation '*par*' indicates the varied parameter. In the upper two panels, parameters *q*, σ_*cap*_, [*Cp*]_*ec*_ and *τ* *_CpFa,ec_* indicate plasma flow, capillary length dispersion, *Cp* concentration in *ec* compartment and dissociation time constant of *CpFa* in *ec* compartment, respectively. In the middle two panels, symbols *τ* *_CpFa,cap_*, *τ*_*CpFa,is*1_, and *τ* *_CpFa,myo_* indicate dissociation time constants of *CpFa* in the compartments *cap*, *is*1 and *myo*, respectively. Symbols [*Cp*]_*is*1_ and [*Cp*]_*myo*_ indicate total *Cp* concentration in the compartments *is*1 and *myo*, respectively. To facilitate judgment of timing with the dilution curves, the *Fa*-curves from Fig 4A are shown in the bottom panels. Note that in the middle left panel scaling is different from the panel above.

Since all investigated parameters are positive, and *Fa* concentrations are positive too, we quantified sensitivity as a ratio of relative changes. For the sensitivity analysis, the time course of normalized *Fa* ^*L*^_*tot*_ concentration [*Fa* ^*L*^_*tot*_](*t*), as presented in Fig 4A, is used as reference curve, a copy of which is shown in the lower panels of Fig 5 to facilitate judgment of timing. In Fig 5, sensitivity functions are presented in a format similar to Kellen and Bassingthwaighte [38]. For each investigated parameter *par*, simulations of time dependent concentrations of [*Fa* ^*L*^_*tot*_](*t*) were carried out for a high value of *par*_*hi*_ = *par*×1.2 and a low value of *par*_*lo*_ = *par*/1.2. To describe the relative sensitivity of the tail *SFa*_*tail*,*par*_(*t*) of the *Fa* washout curves we used the following expression and its numerical approximation:

$$SFa_{tail,par}\left(t\right)=\frac{par}{Fa^{L}{}_{tot}}\frac{\partial \;Fa^{L}{}_{tot}\left(t\right)}{\partial \;par}\approx \frac{\text{ln}\left(Fa^{L}{}_{tot}\left(t,par_{hi}\right)/Fa^{L}{}_{tot}\left(t,par_{lo}\right)\right)}{\text{ln}\left(par_{hi}/par_{lo}\right)}$$ (14)

For example, if [*Fa* ^*L*^_*tot*_](*t*) would be proportional to the value of *par*, tail sensitivity would be equal to 1 according to Eq (14). Note that in deriving the numerical approximation of relative sensitivity (right term Eq 14) the general rule is used that the logarithm of a quotient equals the difference between the logarithms of nominator and denominator. The expression for tail sensitivity appears not suited to describe sensitivity around the peak of the washout curve, because before the onset of the peak, concentrations *Fa* ^*L*^_*tot*_(*t*,*par*_*hi*_) and *Fa* ^*L*^_*tot*_(*t*,*par*_*lo*_) both equal zero. Therefore, for sensitivity of the peak *SFa*_*peak*,*par*_(*t*) of the washout curves and its numerical approximation we used:

$$SFa_{peak,par}\left(t\right)=par\frac{\partial \;Fa^{L}{}_{tot}\left(t\right)}{\partial \;par}\approx \frac{Fa^{L}{}_{tot}\left(t,par_{hi}\right)-Fa^{L}{}_{tot}\left(t,par_{lo}\right)}{\text{ln}\left(par_{hi}/par_{lo}\right)}$$ (15)

The value 1.2 for the factor of variation of *par* was chosen as a compromise so that the effect was sufficiently large for good accuracy and not too large to be hindered by non-linearity. For all parameter changes, the time course of *Alb* ^*L*^ concentration remained in perfect fit, but, as expected, the time course of [*Fa* ^*L*^_*tot*_] altered.

The dilution curves are actually determined by two independent parameters per compartment, *i*.*e*., storage capacity and boundary permeability. Thus within the compartments, effects of parameter variations are mutually dependent. Using the analytical expressions in Eq (13) for storage capacity and in Eq (6) for boundary permeability, the sensitivity to variations in volume fraction *V*_*c*_/*V*_*all*_, membrane area *S*_*mem*_, diffusion coefficient *D*_*Fa*_, and equilibrium constant *K*_*CpFa*_ in the four compartments can be estimated from the presented sensitivities *SFa*_[Cp]_ to [*Cp*] and *SFa*_*τCpFa*_ to *τ*_*CpFa*_ for the corresponding compartments. Thus, the dependent relative sensitivities on the left are written as a function of the calculated sensitivities on the right

$$\begin{array}{l} SFa_{Vc}\approx SFa_{\left[Cp\right]}+SFa_{\tau CpFa}\\ SFa_{Smem}\approx 2\;SFa_{\tau CpFa}\\ SFa_{KCpFa}\approx -SFa_{\left[Cp\right]}\\ SFa_{DFa}\approx SFa_{\tau CpFa} \end{array}$$ (16)

The effect of membrane uptake of *Fa* from the *CpFa* complex by direct contact with the membrane is governed by the membrane reaction rate parameter *d*_*Cp*_. The ratio *d*_*Cp*_/*d*_*Fa*_ determines its relative contribution to boundary permeability *P*_*b*_ according to Eq (6). To facilitate estimation of the latter contribution, *d*_*Fa*_ has been calculated with Eq (6) for all compartment-membrane boundaries.

In Fig 5, parameter sensitivity of the *Fa* ^*L*^ dilution curve is presented graphically. The left panels focus on peak sensitivity, and the right panels on tail sensitivity. To show how to read these graphs, as an example we present a change in concentration [*Cp*]_*ec*_ in recipe style. Increasing the concentration [*Cp*]_*ec*_ by, for instance, 10% results in an equivalent increase of the logarithm by ln(1.1), *i*.*e*., 0.095. Next, the curves for peak and tail sensitivity to [*Cp*]_*ec*_ in the left and right panels, respectively, are scaled vertically by this factor of 0.095. The final result, not shown in the figure, is obtained by adding the latter scaled curves to the reference curves, shown in the lower panels. In this example, the peak in the left panel lowers and the tail in the right panel elevates. The most pronounced sensitivities are associated with parameters *q*, *σ*_*cap*_, *τ*_*CpFa*,*ec*_ and [*Cp*]_*ec*_ for the peak and parameters *q*, *τ*_*CpFa*,*ec*_, [*Cp*]_*ec*_ and [*Cp*]_*myo*_ for the tail. Capillary length dispersion *σ*_*cap*_ was assumed to be 0.5. Increase of *σ*_*cap*_ (upper panels) results in elevation of the upslope and slight depression of the downslope of the peak and the tail of the *Fa* ^*L*^ dilution curve. Although flow *q* is experimentally determined, we analyzed the related sensitivity of the *Fa* ^*L*^-curve because of its potential importance. Elevation of *q* causes a rise of the *Fa* peak and depression of the tail, implying that less *Fa* enter the myocardial tissue during the peak phase, probably because less time is available for exchange. An increase of the value of dissociation time constant *τ*_*CpFa*,*ec*_ of the endothelial *CpFa* complex (upper panels), *i*.*e*., a lower *FABP*-*Fa* dissociation rate, results in elevation of the peak and suppression of the tail, indicating less uptake and less back flux. For all other compartments, sensitivity to the time constant *τ*_*CpFa*_ is considerably less than in the *ec*-compartment. The sensitivities to changes in *τ*_*CpFa*,*ec*_ and [*Cp*]_*ec*_ appeared to be opposite and equal in amplitude. For the *myo* compartment, the effect of varying *τ*_*CpFa*_ is negligible relative to that of [*Cp*], especially in the tail, suggesting that for this compartment storage function is of major importance.

In Table 3 the calculated values for permeability (Eqs 6–8) and storage capacity (Eq 13) are presented for the three experimental conditions shown in Fig 4. Permeability values *P*_*b*_, related to binding kinetics of *Fa* and *Cp* to form *CpFa* near the phospholipid membranes, appear to be considerably lower than the permeability values *P*_*aqu*_ related to *Fa* transfer through the aqueous compartments. Furthermore, for the *Fa* concentration profiles in all compartment boundary zones, the exponential decay distance constants *d*_*Fa*_ (Eq 6) are presented. According to Eq (6), the ratio of permeability by the contact pathway and the detach pathway equals *d*_*Cp*_/*d*_*Fa*_. Using *d*_*Cp*_ = 5 nm (Table 2) results in values of *d*_*Cp*_/*d*_*Fa*_ of 0.86, 0.05, 0.72 and 0.31 for the boundary layers near the membranes encapsulating the compartments *cap*, *ec*, *is*1 and *myo*, respectively, for the experiment simulated in Fig 4C. In this specific experiment the conditions are close to the normal physiological conditions with respect to albumin and *Fa* concentrations.

**Table 3:** Permeability and storage capacity of compartments and membranes.

Compartment	*P*_*aqu*_	*P*_*b*_	*V*_*Fa*,*c*_ /*V*_*all*_	*d*_*Fa*_	Membrane	*P*_*bmemb*_	*V*_*Fa*,*mem*_/*V*_*all*_
	m s^-1^	m s^-1^	-	nm		m s^-1^	-
**A. [*Alb*] = *0*.*11mmol l*** ^***-1***^, **[*Fa***_***tot***_ **] = *0*.*10 mmol l*** ^***-1***^ (Exp.3005612c)							
*cap*	2.38	0.0655	2613	11.5			
					*cap*,*ec*	0.0055	300
*ec*	0.36	0.0060	8.1	90.4			
					*ec*,*is*1	0.0054	328
*is*1	10.5	0.0543	395	13.3			
					*is*1,*myo*	0.0238	356
*myo*	1.08	0.0424	12076	14.9			
**B. [*Alb*] = *0*.*0147mmol l*** ^***-1***^, **[*Fa***_***tot***_ **] = *0*.*0133 mmol l*** ^***-1***^ (Exp.070367a)							
*cap*	0.363	0.0201	372	30.5			
					*cap*,*ec*	0.0047	300
*ec*	0.415	0.0062	8.6	87.5			
					*ec*,*is*1	0.0045	328
*is*1	1.608	0.0170	56.2	35.2			
					*is*1,*myo*	0.0123	356
*myo*	1.253	0.0271	13034	14.3			
**C. [*Alb*] = *0*.*44mmol l*** ^***-1***^, **[*Fa***_***tot***_ **] = *0*.*40 mmol l*** ^***-1***^ (Exp.0703610d)							
*cap*	9.08	0.1678	10210	5.8			
					*cap*,*ec*	0.0056	300
*ec*	0.31	0.0057	7.4	94.3			
					*ec*,*is*1	0.0055	328
*is*1	31.5	0.0130	1450	6.9			
					*is*1,*myo*	0.0298	356
*myo*	0.78	0.0386	10316	16.1			

Symbols *cap*, *ec*, *is*1 and *myo* indicate capillary, endothelial, pericapillary interstitial and cardiomyocyte compartments, respectively. Symbols *P*_*aqu*_, *P*_*b*_ and *P*_*bmemb*_ indicate permeability of aqueous compartments, the aqueous boundary zones near a phospholipid membrane and the membranes including the boundary zones on both sides, respectively. Ratios *V*_*Fa*,*c*_/*V*_*all*_ and *V*_*Fa*,*mem*_/*V*_*all*_ indicate storage capacity of compartments and membranes, respectively, as normalized to summed volume of tissue and fluid compartments. Symbol *d*_*Fa*_ indicates exponential decay distance constant for the *Fa* concentrations in the boundary zones near the membrane (Eq 6). Letters A, B and C refer to the corresponding panels in Fig 4.

The volume ratio *V*_*Fa*,*c*_/*V*_*all*_, as calculated with Eq (13), represents storage capacity of compartment *c* normalized to total volume of tissue and fluid. Because the equilibrium constant *K*_*CpFa*_ is very low relative to the physiological [*Cp*], the ratio *V*_*Fa*,*c*_/*V*_*all*_ is generally much larger than 1, even if compartment volume is small. The storage capacity of a membrane is calculated as the product of membrane volume and *Fa* partition coefficient of membrane lipid relative to that of water. From Table 3 we concluded that by far most of the storage capacity is located in the capillary lumen and in the cardiomyocytes. Furthermore, storage capacity of the membranes cannot be neglected relative to that of the relatively small *ec* and *is*1 compartments.

## Discussion

We have developed a novel model to quantify *Fa* transfer through the coronary system, and their uptake into and transfer in the surrounding myocardial tissue, primarily based upon common physicochemical and physiological principles (Figs 1 and 2). In this model *Fa*, bound to albumin, move from arteries into capillaries. Along the capillaries, part of the *Fa* is released from albumin, permeates the endothelial cell and pericapillary interstitial compartment, reaching the cardiomyocyte interior where *Fa* are metabolized. The remainder of the capillary *Fa* is transferred downstream to the venous effluent by convection.

Although the applied physiological and physicochemical principles, forming the basis of our model, are classical, integration of these principles into a complete physiological model subsystem is novel. To the best of our knowledge, there is no cardiac model available, taking into account *Fa* transfer from capillary to cardiomyocyte crossing three membranes, while considering *Cp*-mediated diffusion of *Fa* in the aqueous cellular and extra-cellular compartments in between. A major breakthrough is the novel analytical solutions of mathematical equations describing how soluble carrier proteins *Cp* facilitate diffusion of *Fa* through the aqueous compartments and how *Fa* transfer is hampered by the rate-limiting process of *Fa* binding to and release from the *Cp* on both sides of the cellular phospholipid membranes. The model handles time dependency of *Fa* concentrations, allowing simulation of multiple tracer dilution experiments and, hence, comparison with the outcome of these experiments on the intact heart. Many of the parameters related to ultrastructural geometry, diffusion properties and *Fa* binding to *Cp* were obtained from earlier published findings. A sensitivity analysis is presented to get insight into the consequences of variations in the parameters used in the model. Also, the model outcome allows for comparison with multi-indicator dilution experiments. These unique features of the model facilitate studies on the complex process of *Fa* uptake by the heart.

The set of differential equations that describes the transfer of *Fa* through a water-membrane interface, incorporating effects of binding dynamics between *Cp* and *Fa* in the aqueous boundary zone (Eqs 17–23) has been reported before and was solved numerically [19]. Since so many parameters appear to be relevant and dependencies are non-linear, a convenient sensitivity analysis is hard to obtain numerically. Therefore, we used a comprehensive analytical solution (Eqs 6, 8, 23 and 29) for these equations. For a sensitivity analysis, analytical solutions are preferred over numerical solutions, since dependencies on the various parameters are shown directly.

In modeling capillary exchange with the tissue, we introduced the capillary unit as an extension of the Krogh cylinder [39]. The Krogh cylinder was modified by incorporating dispersion of capillary lengths and the exchange of *Fa* with adjacent capillary units. In the example of a hexagonal distribution of capillary cross-sections as shown in Fig 2, a cardiomyocyte is surrounded by three capillaries.

In our model, axial diffusion has been ignored. The error involved in ignoring this diffusion is most likely small, because the average capillary transit time by convection is in the order of 10 s under physiological conditions of blood perfused hearts *in situ*. During that time, the diffusion front spreads by the square root of the product of convection time and the diffusion coefficient of albumin. Using the data presented in Table 2, we find for this distance spread 30 μm, which is far less than the 800 μm, being the average capillary length in the heart [14]. Furthermore, it can be derived that the fractional contribution of axial diffusion relative to axial convection for arterio-venous transport in the capillaries equals the diffusion coefficient divided by the product of flow velocity and mean capillary length, which fraction is only 10^−3^. Note that in our experiments on isolated, saline perfused hearts the transit time is only about 2 s (Fig 4), implying that for that situation the effect of axial diffusion is even less.

Consensus exists about the pivotal role of cytoplasmic *FABP* in transferring *Fa* from the inner phospholipid leaflet of the cardiac cell membrane to the main site of metabolic conversion in the cardiomyocyte, *i*.*e*., the mitochondrion. The absence of cytoplasmic *FABP* resulted in a substantial decline in *Fa* utilization in both intact hearts *in situ* [40] and isolated cardiomyocytes [41]. Moreover, the high concentration of *FABP* in the cardiomyocyte strongly suggests a role of *FABP* in carrier-mediated *Fa* diffusion [11]. Alternatively, conversion of *Fa* into acylCoA in or close to the cellular membrane, *i*.*e*., vectorial acylation [42, 43], may create a cytoplasmic sink for *Fa* and, hence, promote *Fa* uptake due to a steeper concentration drop across the cellular membrane. At present, there is no solid proof that this mechanism quantitatively plays a role in cardiac *Fa* uptake. Therefore, this membrane-protein catalyzed process is not included in our model.

Often, cell membranes are considered to be the major hindrance for *Fa* transfer [7, 15, 16] within organs. Considering the membrane as a lipophilic material with a thickness of 5 nm, a partition coefficient of *C*_*lipid*_ = 9∙10^5^ [8] and an estimated diffusion coefficient *D*_*Fa*_ for free *Fa* similar to that in water (5∙10^-10^ m^2^s^-1^, [24]), membrane permeability *P*_*mem*_ = *C*_*lipid*_ *D*_*Fa*_/*d*_*mem*_ would be extremely high, *i*.*e*., 9∙10^4^ m s^-1^, as compared to the permeability data presented in Table 3. Transfer of *Fa*, however, could be hindered by interactions of the lipophilic compounds with the head-groups of the phospholipid bilayer, limiting stable positioning of *Fa* either to the inside or to the outside of the membrane. While permeating the membrane, the *Fa* molecule is likely to be hindered considerably by the parallel organized phospholipids. Accordingly, the diffusion coefficient is diminished to 4∙10^−12^ m^2^s^-1^ [44], resulting in a membrane permeability of 700 m s^-1^, which is still very high relative to the permeability of the boundary layers on both sides of the membrane, as shown in Table 3. In accordance with the insights reported by Hamilton [45], using known physicochemical principles, we found that the most important hindrance to *Fa* transfer should not be attributed to the phospholipid membranes, but to the boundary permeability (Fig 3), representing diffusion of free *Fa* through the thin aqueous boundary zone from the location of *Fa* release from *Cp* towards the surface of the phospholipid membrane, or *vice versa*.

We cannot exclude that a putative, so-called membrane-protein transporter is 'hidden' in one of the model parameters. The following concept has to be considered. In our model *Fa* are transferred from the soluble *Fa*-carrier *Cp* to the endothelial membrane through the aqueous boundary zone near the membrane by two pathways, *i*.*e*., the detach pathway and the contact pathway (Fig 3A). In this vision, the contribution of such a transport protein could be enhancement of the contact pathway by facilitation of the transfer of *Fa* between *Cp* and phospholipid membrane. We did not use the more general term 'transport protein', because of its frequent use in relation to active or directional transport mechanisms, but the term Transfer Facilitating Membrane Protein (*TFMP*) instead. Such a protein could be CD36 [46]. The rate parameter for contact pathway transfer is quantified by the membrane reaction rate parameter *d*_*Cp*_. Our model predicts that *Fa* uptake from the capillary compartment to the interior of the cardiomyocyte is feasible without the involvement of *TFMP*. However, it should be realized that the latter model outcome relates to the loading conditions of the heart, as present in the multiple indicator dilution experiments of the rabbit heart perfused *in vitro*. It cannot be excluded that for higher mechanical loading conditions, *TFMP* may be needed to guarantee sufficient *Fa* transfer as indicated by Bonen and colleagues [47]. The effect of *TFMP* is described quantitatively by an increase of the reaction rate parameter *d*_*Cp*_ relative to the value attributed to the *TFMP*-free phospholipid membrane.

Clefts between the endothelial cells have been suggested to facilitate transport of compounds from the *cap* to the *is*1 compartment. For *Fa* transfer, diffusion of free *Fa* through the clefts can likely be ignored, because the cross-section of these channels is less than 1‰ of the surface area of the endothelial membrane, while the tortuous cleft channels are substantially longer than the thickness of the endothelial aqueous boundary layers available for free *Fa* diffusion from the capillary lumen to *is*1 [48, 49]. Facilitation of diffusion through the clefts by albumin is also unlikely, because the albumin transfer through the clefts, if any, takes place in a different time domain because of its large molecular size [3].

In Table 3 permeability of the *is*1-*myo* membrane, *i*.*e*., the sarcolemma, ranges from 1.2 to 3.0 cm s^-1^, which agrees quite well with the value of 1.61 cm s^-1^, as determined by Kamp and Hamilton [17]. Permeability of the endothelial membranes was calculated to be appreciably lower than that of the sarcolemma, due to the low concentration of *FABP* in the endothelial cells as reported by van Nieuwenhoven and coworkers [30]. Furthermore, the permeability of the aqueous compartments (*P*_*aqu*_) is notably higher than that of the corresponding boundary zones (*P*_*b*_), indicating that diffusion of *CpFa* is not substantially hindered in the bulk region of the compartments. By comparing the various compartments, our calculations suggest that the endothelium represents the major resistance to diffusion as has been suggested earlier [50]. Quantitative data on endothelial diffusion properties are relatively inaccurate because its *Cp* concentration cannot be easily measured in such a small compartment. Experimental data suggest that *FABP* fulfill the role of endothelial *Cp*, the concentration of which being probably on the order of 7 μmol l^-1^ [30], a value substantially lower than the *FABP* concentration in cardiomyocytes. It should be noted, however, that even this very low concentration of *FABP* in the endothelial cell is sufficiently high to permit *Fa* transfer though the endothelial cytoplasm at a physiologically relevant level.

Little is known about the dissociation time constant *τ*_*CpFa*,_ of the *CpFa* complex in the various compartments. With the stopped flow technique, the time constant for the albumin complex was reported to be 83 ms [27], but this value is only a maximum value for this time constant, since the technique was not appropriate to measure faster dissociations. The time constants for other *Cp* than albumin are unknown. In our model the value for albumin was assumed to hold for all *Cp* used.

The storage capacity is substantial in those compartments where both *Cp* concentration and volume are sufficiently high, *i*.*e*., in the capillary lumen and the cardiomyocyte. The values for the *Cp* concentrations, obtained from earlier published data, are summarized in Table 2. Despite the high *Cp* concentration in *is*1 (albumin), its storage capacity is small due to its small volume. The volume of the remaining part of the interstitium, *i*.*e*., the non-pericapillary *is*2, is larger, making that compartment more relevant for *Fa* storage. The volumes of the various compartments were recently measured in rabbit hearts, and the values are summarized in Table 1. Because of its remote location relative to the capillary and its close contact with the cardiomyocytes, in the model, the *is*2 storage capacity was added to that of the cardiomyocytes. The remote location of *is2* makes this compartment less relevant for transfer of *Fa* from capillary to cardiomyocyte. Membranes can store *Fa* too, because of its high partition coefficient. Membrane volume, however, is small. Therefore, its storage capacity is relevant only in comparison with the storage capacity of other small compartments, such as *ec* and *is*1 (Table 3).

In analyzing multiple indicator dilution experiments, we estimated the time course of the albumin concentration at the entrance of the capillaries from the measured output concentration, using deconvolution in time [31]. Normally, deconvolution is sensitive to noise, often resulting in large variations in the solution. The convolution technique improved considerably by using the obvious property that concentration cannot be negative and using a weight factor for smoothing by minimization of the second derivative. Although some oscillations may occur, the thus found solution for deconvolution of albumin concentration appeared to be stable and unique. In a test, we lowered the smoothing parameter by a factor of 10, resulting in considerable oscillations in the result of deconvolution. Although the deconvolution became noisy, it is noteworthy that the simulated *Fa* dilution curve as obtained from the venous effluent did not change by more than ±2% over the whole range of the dilution curve.

### Conclusion

To the best of our knowledge, we designed and tested for the first time a model of long-chain fatty acid (*Fa*) transfer through the coronary system and from coronary capillary to the cardiomyocyte that can handle dynamic changes in *Fa* concentration. Capillary lumen, endothelial cell interior, pericapillary interstitium and the cardiomyocyte interior were handled as compartments. The phospholipid membranes separating these compartments were considered to be passed by diffusion of free *Fa*. Delivery of *Fa* to the cellular membrane surface can be achieved by two separate pathways: the detach pathway, *i*.*e*., diffusion of free *Fa*, and the contact pathway, *i*.*e*., the transfer of *Fa* from *CpFa* to the membrane by direct contact. Applying standard physicochemical principles of convection, diffusion and reaction kinetics of *Fa* binding to compartment-specific carrier proteins, the transfer of *Fa* from capillary to cardiomyocyte appeared to be within the experimentally determined range, at least under the present experimental conditions. Although this *Fa* transfer was achieved without the support of auxiliary proteins attached to or residing inside the cellular membrane, it cannot be excluded that the involvement of such proteins is required under higher cardiac loading conditions and, hence, an increased need for *Fa* as energy-rich substrates. Their facilitating action is covered by the model parameter *d*_*Cp*_, increasing the contribution of the contact pathway in overall *Fa* transmembrane transfer. Three multiple tracer dilution experiments with labeled albumin and labeled *Fa*, carried out in the isolated rabbit heart, were simulated successfully. We conclude that the present computational model is a useful tool to obtain quantitative information about cardiac uptake and intramyocardial transfer and storage of blood-borne *Fa*.

## Materials and Methods

### Ethics statement

The experiments with the rabbit hearts were performed in accordance with Guide for Care and use of laboratory animals published by the US National Institute of Health (NIH publication Nr 85–23).

### Permeability of a water-phospholipid membrane boundary for *Fa*

In transferring *Fa* across the boundary from an aqueous fluid compartment into the phospholipid bilayer of a cellular membrane, the complex *CpFa* reaches the boundary zone by radial diffusion (Fig 3). A small fraction of *CpFa* dissociates to form free *Fa*. Approaching the boundary, transfer of *Fa* occurs increasingly by diffusion of free *Fa* at the cost of the fraction carried by carrier-mediated diffusion. Since *Fa* disappear from the aqueous compartment at the boundary, free *Cp* moves back from the boundary zone into the aqueous bulk. Besides this so-called detach pathway we consider the contact pathway, in which the complex *CpFa* delivers *Fa* by direct physical contact to the phospholipid membrane. First, we will derive the equations, describing transfer of *Fa* through the boundary on the basis of the detach pathway only. Thereafter, we add effects of the parallel contact pathway through direct contact of *CpFa* with the respective membrane.

### Transfer of *Fa* through a water-phospholipid membrane boundary with the detach pathway only

Transfer of compounds through a boundary surface is generally quantified by flux *φ* in [mol m^-2^s^-1^]. If we assume that a total *Fa* flux *φ*_*Fa*,*tot*_ is forced to permeate the boundary into the phospholipid bilayer in steady state, then, at all distances *x* from the boundary, flux *φ*_*Fa*,*tot*_ is constant and equal to the sum of complex *Fa* flux *φ*_*CpFa*_ and free *Fa* flux *φ*_*Fa*_:

$$\varphi_{Fa,tot}=\varphi_{CpFa}\left(x\right)+\varphi_{Fa}\left(x\right)$$ (17)

Note that coordinate *z* has been used for axial distance from the capillary entrance, and that coordinate *x* is perpendicular to *z*, pointing in the direction perpendicular to the membranes between the compartments. For the derivation below we assume stagnancy of the fluid near the membrane boundary. Assuming that concentrations at a given location do not change with time, an increase of *CpFa* flux along distance coordinate *x* implies that *CpFa* is formed at that location by binding of *Fa* to *Cp*. This phenomenon is described by the following relation between the spatial gradient of flux *φ*_*CpFa*_ and the local rate of *CpFa* formation:

$$\frac{d\varphi_{CpFa}\left(x\right)}{dx}=\frac{\left[Cp\right]\left(x\right)\;\left[Fa\right]\left(x\right)-\left[CpFa\right]\left(x\right)\;K_{CpFa}}{K_{CpFa}\;\tau_{CpFa}}$$ (18)

Symbols *K*_*CpFa*_ and *τ*_*CpFa*_ indicate equilibrium constant and dissociation time constant of the binding reaction of *Fa* to *Cp*, respectively. Note that with continuous formation of *CpFa* there is no chemical equilibrium, implying that the right term of Eq (18) is not equal to zero. For free *Fa*, free *Cp* and complex *CpFa*, flux depends on the location *x* relative to the membrane, the concentration gradient and the diffusion coefficients *D*_*Fa*_ and *D*_*Cp*_:

$$\varphi_{Fa}\left(x\right)=-D_{Fa}\frac{d\left[Fa\right]\left(x\right)}{dx}$$ (19)

$$\varphi_{CpFa}\left(x\right)=-D_{Cp}\frac{d\left[CpFa\right]\left(x\right)}{dx}$$ (20)

$$\varphi_{Cp}\left(x\right)=-D_{Cp}\frac{d\left[Cp\right]\left(x\right)}{dx}$$ (21)

Diffusion coefficients of free *Cp* (*D*_*Cp*_) and the complex *CpFa* are considered to be the same, because mobility of the large *Cp* is assumed not to be influenced by carrying the relatively small *Fa* molecule. Considering the detach pathway (Fig 3) only, *CpFa* flux equals zero at the boundary, because the complex cannot enter the membrane at that location:

$$\varphi_{CpFa}(0)=0$$ (22)

Using the software package Mathematica 7 (Wolfram Research), we solved Eqs (17–22) analytically, while neglecting the terms in flux *φ*_*Fa*,*tot*_ of the order of 2 and higher. For [*CpFa*] as a function of distance *x* from the boundary, we found:

$$\left[CpFa\right]\left(x\right)=\left[Cp_{tot}\right]-{\left[Cp\right]}_{0}+\frac{\varphi_{Fa,tot}\left(-x+d_{Fa}e^{x/d_{Fa}}\right)}{D_{Cp}\left(1+\alpha \right)}$$ (23)

with $\alpha =\frac{D_{Fa}K_{CpFa}\left[Cp_{tot}\right]}{D_{Cp}{\left[Cp\right]}_{0}{}^{2}}$

and $d_{Fa}\approx \sqrt{\frac{D_{Fa}K_{CpFa}\;\tau_{CpFa}}{{\left[Cp\right]}_{0}}}$

Total *Cp* concentration [*Cp*_*tot*_] is constant and equal to the concentration in the aqueous compartment. Concentration [*Cp*]_0_ indicates the concentration of free *Cp*, as extrapolated linearly from the concentration profile in the aqueous bulk to the boundary. Since gradients in concentration *CpFa*(*x*) are small (Fig 3), and [*Cp*_*tot*_] is constant, gradients in the difference [*Cp*](*x*) are small too. Therefore, in Eq (23), as a first approximation, we assumed a single value [*Cp*]_0_ to be representative of [*Cp*](*x*) in general. Variable *α* represents the ratio of *Fa* transfer by diffusion of free *Fa* relative to transfer by carrier-mediated diffusion far from the membrane boundary. Commonly, *α* is much smaller than 1, e.g., in the capillary plasma *α*<0.002. Variable *d*_*Fa*_ represents the distance constant of exponential decay in the boundary zone. The solution for fluxes and concentrations as a function of *x* is presented graphically in Fig 3, panels B-D.

### Effect of *Fa* delivery to the phospholipid membrane by the contact pathway

As indicated above, by neglecting the contact pathway (Fig 3) for *Fa* transfer, complex flux *φ*_*CpFa*_(0) equals zero at the boundary according to Eq (22). By allowing *CpFa* to deliver *Fa* to the membrane by direct physical contact, we introduce a new boundary condition, located at *x*_b,_ so that we can still use the solution of the differential equations as presented above. At this new boundary, *CpFa* delivers free *Fa* to the phospholipid surface, while free *Cp* returns into the aqueous boundary zone. Thus, *CpFa* flux is converted to an equal free *Fa* flux into the membrane and an equal free *Cp* flux in backward direction. In the state of equilibrium without *Fa* flux, both the detach pathway and the contact pathway bear no flux, because flux cannot be circular without adding external energy. In the presence of *Fa*-flux, at boundary *x*_*b*_, *CpFa* flux is proportional to the deviation from the state of equilibrium. Thus Eq (22) is replaced by the following boundary equation, using Eq (18) to define the deviation from the state of zero flux equilibrium:

$$\varphi_{CpFa}\left(x_{b}\right)=\;d_{Cp}\;\left(\frac{\left[Fa\right]\left(x_{b}\right)\;\left[Cp\right]\left(x_{b}\right)-K_{CpFa}\left[CpFa\right]\left(x_{b}\right)}{K_{CpFa}\;\tau_{CpFa}}\right)$$ (24)

Note that membrane reaction rate parameter *d*_*Cp*_ represents the product of *τ*_*CpFa*_ and the ratio of *Fa* flux due to *CpFa* dissociation at the membrane surface and the *CpFa* concentration in the aqueous boundary zone in virtual absence of any free *Fa* in the membrane. Parameter *d*_*Cp*_ appears to have the physical dimension of a distance, expressing membrane dissociation rate versus bulk dissociation rate of *CpFa*. Now we search for a distance *x*_*b*_ in the solution as represented by Eq (18), so that the boundary condition in Eq (24) is satisfied exactly. Substitution of Eq (18) in Eq (24) yields

$$\varphi_{CpFa}\left(x_{b}\right)=\frac{d\varphi_{CpFa}\left(x_{b}\right)}{dx}d_{Cp}$$ (25)

Substitution of the *x*-derivative of Eq (23) in Eq (20) yields

$$\varphi_{CpFa}\left(x\right)=\varphi_{Fa,tot}\frac{1-e^{x/d_{Fa}}}{1+\alpha }$$ (26)

Substitution of *φ*_*CpFa*_(*x*) obtained from Eq (26) into the left and right term of Eq (25) renders a solution for *x*_b,_ so that the boundary condition Eq (24) is satisfied:

$$x_{b}=-d_{Fa}\text{ln}\left(1+d_{Cp}/d_{Fa}\right)$$ (27)

Note that if *d*_*Cp*_<<*d*_*Fa*_, approximation *x*_*b*_ ≈ -*d*_*Cp*_ may be used. In Fig 3, related solutions for concentrations [*CpFa*](*x*) and [*Fa*](*x*) and fluxes *φ*_*CpFa*_(*x*) and *φ*_*Fa*_(*x*) are shown graphically. For zero flux *φ*_*Fa*,*tot*_, [*Fa*] equals the equilibrium concentration for the given [*Cp*_*tot*_] and [*Cp*]_0_, resulting in [*Fa*]_*equ*_ = *K*_*CpFa*_([*Cp*_*tot*_]/[*Cp*]_0_−1). In the presence of *Fa*-flux, [*Fa*] depends on *x* by adding to the equilibrium concentration a term proportional with flux *φ*_*Fa*,*tot*_. The proportionality factor is found by integration of flux *φ*_*Fa*_(*x*) according to Eq (19), while using *φ*_*Fa*_(*x*) = *φ*_*Fa*,*tot*_—*φ*_*CpFa*_(*x*) according to Eq (17). The solution for flux *φ*_*CpFa*_(*x*) is presented in Eq (26). Thus for concentration [*Fa*](*x*), it is derived:

$$\left[Fa\right]\left(x\right)=k_{CpFa}\;\left(\frac{\left[Cp_{tot}\right]}{{\left[Cp\right]}_{0}}-1\right)-\varphi_{Fa,tot}\frac{d_{Fa}e^{x/d_{Fa}}\;+\alpha \;x}{\left(1+\alpha \right)D_{Fa}}$$ (28)

Permeability *P* of a layer with thickness *h* equals the ratio of flux divided by the *Fa*-concentration drop from point *x* = *-h+x*_*b*_ to point *x* = *x*_*b*_. For a given flux *φ*_*Fa*,*tot*_ with Eq (28), [*Fa*] is calculated for *x* = *x*_*b*_ and *x* = *x*_*b*_+*h*, being used to calculate the drop of [*Fa*]. Permeability *P* equals flux divided by [*Fa*]-drop, resulting in:

$$P=\frac{1}{1/P_{b}+1/P_{aqu}}$$ (29)

with $P_{b}=\frac{\left(1+\alpha \right)}{\left(1-e^{-h/d_{Fa}}\right)}\frac{D_{Fa}}{d_{Fa}}\left(1+\frac{d_{Cp}}{d_{Fa}}\right)\approx \frac{D_{Fa}}{d_{Fa}}\left(1+\frac{d_{Cp}}{d_{Fa}}\right)$

and $P_{aqu}=\frac{1}{h}\left(D_{Fa}+D_{Cp}\frac{{\left[Cp\right]}_{0}{}^{2}}{\;k_{CpFa}\left[Cp_{tot}\right]}\right)$

Permeability *P*_*b*_ represents the effect of detachment of *Fa* from *CpFa* in a thin aqueous layer in the boundary zone, in close vicinity of the cellular phospholipid membrane, the thickness of which is determined by decay distance constant *d*_*Fa*_ (Eq 23, e.g. in Fig 3 *d*_*Fa*_ = 7 nm). Permeability *P*_*aqu*_ represents the effect of carrier-mediated diffusion in the aqueous layer, excluding boundary effects. The resulting equations for *P*_*b*_ and *P*_*aqu*_ are presented by Eqs (6) and (8), respectively. Often *α*<<1 and h>>*d*_*Fa*_, so that for *P*_*b*_ the approximation can be used as indicated in Eq (29). In the derivation, flux is assumed to be directed from aqueous bulk into the membrane. The derivation is also valid for a negative sign of the flux, implying that calculated permeabilities are also valid for *Fa*-flux from membrane to aqueous compartment, e.g., occurring in the boundary zone on the other side of the membrane.

### Capillary length dispersion

Capillary length dispersion is described by a Gaussian distribution of the logarithm of capillary length *z*, resulting in the probability density function *p*_*len*_(*z*)

$$p_{len}\left(z\right)=\frac{1}{z\;\sigma_{cap}\sqrt{\pi }}\text{exp}\left(-{\left(\frac{\sigma_{cap}}{4}+\frac{\text{ln}\left(z/z_{0}\right)}{\sigma_{cap}}\right)}^{2}\right)$$

with

$$\int_{0}^{\infty }p_{len}\left(z\right)dz=1$$ (30)

Symbols *z*_0_ and *σ*_*cap*_ indicate mean length and relative dispersion, respectively. Assuming that all capillaries have the same diameter, total capillary cross-sectional area *A*_*cap*_(*z*) is proportional to the number of capillaries, longer than length *z*. So, for small *z*, all capillaries are included, implying that *A*_*cap*_(*z*) is maximal, while with increasing *z* ever more short capillaries become excluded, eventually causing *A*_*cap*_(*z*) to decrease to zero. At the capillary entrance, *A*_*cap*_(0) equals total capillary volume *V*_*cap*_ divided by mean capillary length *z*_0_. Thus by backward integration of *p*_*len*_(*z*) it is found:

$$A_{cap}\left(z\right)=\frac{V_{cap}}{z_{0}}\int_{z}^{\infty }p_{len}\left(z\right)dz$$ (31)

Substitution of Eq (30) into Eq (31), followed by determination of the integral, results in Eq (9). Besides *A*_*cap*_, the capillary wall area available for diffusion (*dS*_*cap*_(*z*)/d*z*) is also proportional to the number of capillaries as a function of *z*, resulting in Eq (11). Supposing that the arterio-venous blood pressure drop is the same for all capillaries, we assumed that blood flow velocity is inversely proportional to the length of the capillary. Defining *q*_*tot*_ to be the sum of blood flow in all capillaries at the entrance, for capillary blood flow *q*_*cap*_(*z*) it follows:

$$q_{cap}\left(z\right)=q_{tot}\int_{z}^{\infty }\frac{p_{len}\left(z\right)}{z}dz/\int_{0}^{\infty }\frac{p_{len}\left(z\right)}{z}dz$$ (32)

**Fig 6:**
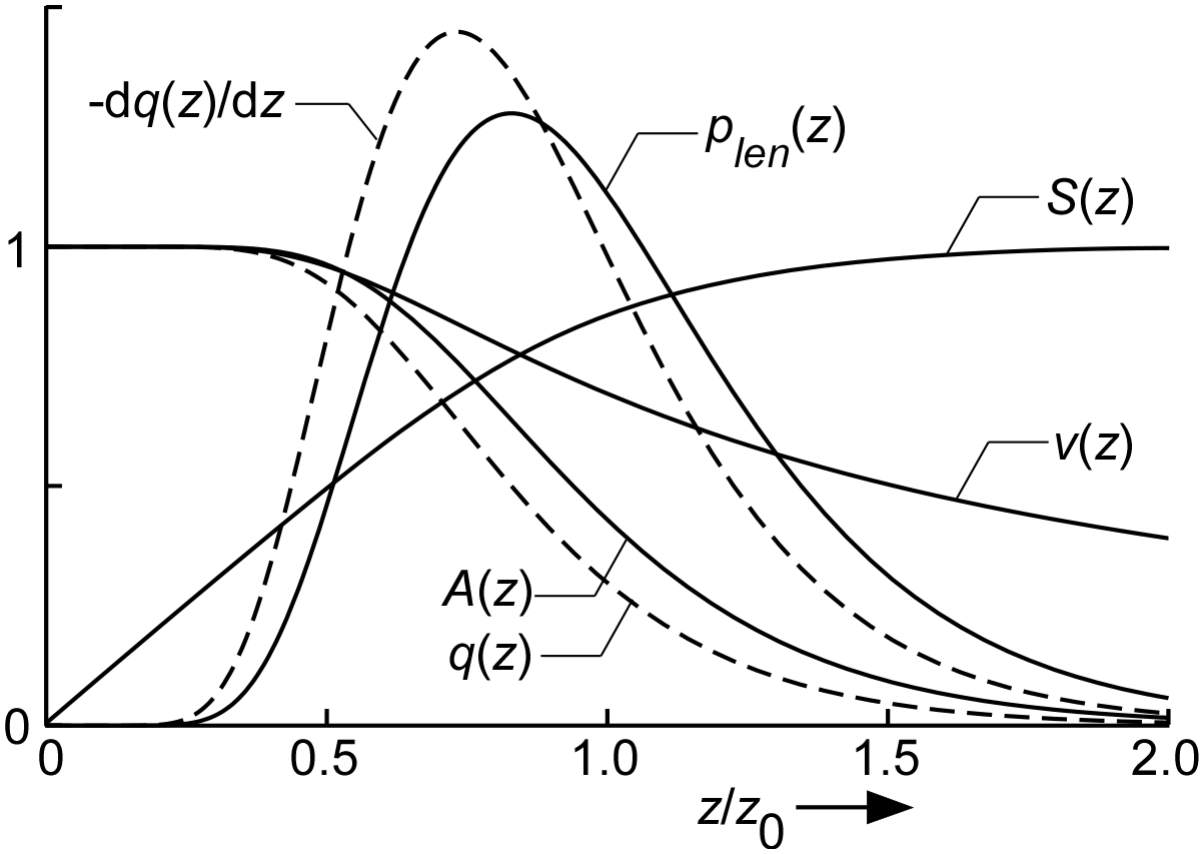
Functions used to incorporate capillary length dispersion. Functions *A*(*z*), *q*(*z*), -d*q*/d*z*(*z*), *v*(*z*) and *S*(*z*) represent normalized capillary cross-sectional area, capillary blood flow, its derivative, mean capillary blood flow velocity and capillary wall area, respectively (Eqs 9–12), as a function of distance from the capillary entrance *z*, normalized to average capillary length *z*_0_. Function *p*_*len*_(*z*) represents capillary length distribution with relative dispersion *σ*_*cap*_ = 0.5 (Eq 30).

Substitution of Eq (30) into Eq (32) results in Eq (10). Mean capillary blood flow velocity *v*(*z*) is calculated as total blood flow *q*_*cap*_(*z*) divided by total cross-sectional area *A*_*cap*_(*z*) (Eq 12). Normalized capillary wall surface *S*(*z*) is found by integration of normalized *A*_*cap*_(*z*) to variable *z*. This function is used in Eq (11). Fig 6 shows the z-dependent functions *p*_*len*_, and the normalized values of *A*_*cap*_, *q*_*cap*_, d*q*_*cap*_/d*z* and *S*_*cap*_, as represented by *A*, *q*, d*q*/d*z* and *S*, for relative dispersion of capillary length *σ*_*cap*_ = 0.5 (Eq 30).

### Parameter substitutions

In the model of *Fa* transfer from capillary to cardiomyocyte, many parameters were obtained from experimental data. Recently, we assessed the myocardial volume fractions of compartments, the surface areas for diffusion and the thicknesses of diffusion layers, as summarized in Table 1 [23]. In solving differential equation Eq (4) with Eq (7), for the scaling constant *S*_*cap*,*tot*_/*V*_*cap*_ in Eq (11) we substituted the experimentally determined ratio *S*_*cap*_/*V*_*cap*_ as can be derived from Table 1. Capillary length dispersion *σ*_*cap*_ in Eqs (9 and 10) was taken to be 0.5. The volume fraction of the compartments is related to total volume *V*_*all*_, being the sum of tissue compartments including all blood compartments. Area *S* is attributed to thin and flat structures. Permeability thickness *h*_*diff*_ of the thin and flat compartments *ec* and *is*1 is representative of the thickness for diffusion. It is determined as the reciprocal of the mean of reciprocals of compartment thickness. Since the *is*2 compartment is flat, we could attribute a mid-compartment surface to it, parallel to the cardiomyocyte membranes on both sides.

In Table 2, physicochemical properties of myocardial constituents are summarized. Most data is obtained from literature. First the values of the general properties are presented, followed by those related to the specific ones per compartment, mostly because type and concentration of *Cp* vary between compartments. Compartments *cap* and *is*1 are combined because in both compartments albumin fulfills the role of *Cp*. For many physicochemical properties estimates were used, because no data is available about the true values. The diffusion coefficient *D*_*Cp*,*cap*_ of albumin in plasma is derived from bovine albumin. Decay time constant *τ*_*CpFa*,*cap*_ of the *Alb-Fa* complex is estimated, but the true value is a matter of debate. About the dissociation time of the other *CpFa* complexes no information is available. The membrane reaction rate parameter *d*_*Cp*_, having the physical dimension of length, is not known for any fluid-membrane interface. For small values of *d*_*Cp*_ relative to *d*_*Fa*_, *d*_*Cp*_ is approximately equal to the virtual distance shift *x*_*b*_ (Eq 27) of the membrane boundary towards the aqueous compartment. As a primary estimate, we assumed the value of this parameter to be equal to the average radius of the *CpFa* complex [36]. The diffusion coefficients of the *Cp* in the *ec* and *myo* compartment are assumed to be twice that of albumin, because their molecular weight is about one quarter of that of albumin. The albumin concentration in the *is*1 compartment is normally about 70–86% of that in the capillary plasma [32]. We used the data of Tschubar *et al* [29], because they found the ratio of 86% to be valid in a large range of arterial *Alb* concentrations (0.033–0.394 mol m^-3^). The *Cp* concentration in the endothelium is known to be less than 4% of the *FABP* concentration in the cardiomyocyte [30], but accurate data is not available. According to our model, the concentration should not be much less, because then the *Cp* concentration may be too low to fulfill the role of carrier protein for *Fa*. However, if the decay time constant of the endothelial *CpFa* complex is smaller than that of albumin in the capillary lumen, the same boundary permeability can be obtained with lower *Cp* concentrations (Eq 6).

## Supporting Information

S1 Data Example of data file containing experimental data. (PDF)

S1 Read Mathematica program to read data file and to convert to Mathematica data. (PDF)

S1 Model Mathematica program, to be executed after reading data. This program follows the analysis as described in the manuscript, finally rendering the Fa concentration as a function of time.
(PDF)
