# Supplementary material for: Modeling Fatty Acid Transfer from Artery to Cardiomyocyte
Source: PLoS Comput Biol. 2015 Dec 16;11(12):e1004666. doi: 10.1371/journal.pcbi.1004666 (PMC4682637; doi:10.1371/journal.pcbi.1004666)
Supplement: S1 Data — (PDF) [file pcbi.1004666.s001.pdf]

```
'TAC file name (Ver.  2.0):'    '3005612.tac'   'REVISED:
27JAN10 10:29:07'
'experiment type:'    'MID'
'experiment description:'  'Unknown'
'experiment name:'    'palm 3005612'
'# of auxiliary data:'       7
'Animal identification:'   'rab'
'Total heart mass (g):'    '8.69440'
'Ventricular mass, LV+RV (g):'  '8.36000'
'Name of drug used:'  'nem'
'Name of drug used:'  'hep'
'Name of drug used:'  ''
'Name of drug used:'  ''
'# of runs:'      1
'run description:'    'Unknown'
'# of auxiliary data:'      24
'Time of day (24h clock):' '0.00000'
'Perfusate type:'     '0.10 mM palm'
'Perfusate flow rate (ml/min):' '16.8000'
'Perfusion pressure (mm Hg):'    '0.300000E-04'
'Perfusate temperature (C):'    '36.5000'
'Perfusate osmolarity:'    ''
'Perfusate Na conc (meq):' '0.00000'
'Perfusate K conc (meq):'  '0.00000'
'Perfusate glucose conc (mg %):''0.00000'
'Hematocrit (%):'     '0.00000'
'Heart rate (BPS):'   '0.126000E-03'
'Injection volume (ml):'   '0.475000'
'Injection duration (sec):'     '0.00000'
'Injection site:'    'ao'
'Sampling site:''rv'
'Support animal BP (mm Hg):'    '0.00000'
'LV vent flow rate (ml/min):'   '1.20000'
'Reference scale factor:'  '1.06838'
'1st diffusible scale factor:'  '0.968990'
'2nd diffusible scale factor:'  '0.968990'
'3rd diffusible scale factor:'  '0.00000'
'4th diffusible scale factor:'  '0.00000'
'1st sampling interval (sec):'  '1.00000'
'2nd sampling interval (sec):'  '4.00000'
'# of physiol datasets:'     0
'# of input function datasets:'   0
'# of sample datasets:'      3
'sample dataset description:'   'Unknown'
'# of lines:'    60
'# of fields:'    4
'time (sec)'    '131I'     '3H'  '14C'
 -2.4000        0.000000000     0.000000000     0.000000000
 -1.4000        0.000000000     0.000000000      0.0000
-0.40000        0.000000000     0.000000000     0.000000000
 0.60000        0.000000000      0.0000         0.000000000
  1.6000        0.44988E-05     0.14087E-05     0.000000000
  2.6000        0.39978E-04     0.33458E-04     0.53070E-04
  3.6000        0.50134E-02     0.30728E-02     0.49515E-02
```

4.6000	0.40098E-01	0.21637E-01	0.35099E-01
5.6000	0.14932	0.76021E-01	0.12456
6.6000	0.22217	0.11264	0.18803
7.6000	0.19664	0.10220	0.16344
8.6000	0.12684	0.73736E-01	0.10678
9.6000	0.84467E-01	0.55341E-01	0.71203E-01
10.600	0.56409E-01	0.47017E-01	0.48713E-01
11.600	0.35096E-01	0.36580E-01	0.30170E-01
12.600	0.21747E-01	0.29760E-01	0.18918E-01
13.600	0.14238E-01	0.25508E-01	0.12911E-01
14.600	0.94998E-02	0.22253E-01	0.91747E-02
15.600	0.68092E-02	0.20081E-01	0.70425E-02
16.600	0.48917E-02	0.17133E-01	0.54639E-02
17.600	0.36640E-02	0.17285E-01	0.44902E-02
18.600	0.28074E-02	0.15866E-01	0.36677E-02
19.600	0.18630E-02	0.14326E-01	0.29614E-02
20.600	0.14847E-02	0.13563E-01	0.25960E-02
21.600	0.13132E-02	0.12928E-01	0.24991E-02
22.600	0.11120E-02	0.12725E-01	0.23319E-02
23.600	0.86307E-03	0.11306E-01	0.20140E-02
24.600	0.82628E-03	0.11249E-01	0.19439E-02
25.600	0.77362E-03	0.10480E-01	0.19076E-02
26.600	0.60070E-03	0.96225E-02	0.18025E-02
31.600	0.35520E-03	0.74643E-02	0.13199E-02
35.600	0.21653E-03	0.59886E-02	0.11639E-02
39.600	0.17561E-03	0.48342E-02	0.98478E-03
43.600	0.13916E-03	0.40340E-02	0.88337E-03
47.600	0.10793E-03	0.36894E-02	0.82919E-03
51.600	0.98044E-04	0.29639E-02	0.73969E-03
55.600	0.97457E-04	0.25953E-02	0.65638E-03
59.600	0.75823E-04	0.23407E-02	0.64919E-03
63.600	0.80109E-04	0.20326E-02	0.56824E-03
67.600	0.52503E-04	0.16671E-02	0.49848E-03
71.600	0.64022E-04	0.14671E-02	0.45639E-03
75.600	0.77297E-04	0.13051E-02	0.43282E-03
79.600	0.50253E-04	0.11490E-02	0.41833E-03
83.600	0.69450E-04	0.10207E-02	0.39287E-03
87.600	0.55920E-04	0.93875E-03	0.35984E-03
91.600	0.61270E-04	0.85686E-03	0.36631E-03
95.600	0.43011E-04	0.71038E-03	0.32284E-03
99.600	0.59805E-04	0.68545E-03	0.31055E-03
103.60	0.39637E-04	0.60762E-03	0.29725E-03
107.60	0.40992E-04	0.52768E-03	0.25663E-03
111.60	0.25629E-04	0.49920E-03	0.23226E-03
115.60	0.42057E-04	0.45052E-03	0.23620E-03
119.60	0.45167E-04	0.39776E-03	0.21433E-03
123.60	0.23934E-04	0.36356E-03	0.19586E-03
127.60	0.43105E-04	0.31256E-03	0.16978E-03
131.60	0.49827E-04	0.30660E-03	0.19506E-03
135.60	0.46675E-04	0.27608E-03	0.18247E-03
139.60	0.24718E-04	0.25661E-03	0.16602E-03
143.60	0.20142E-04	0.22228E-03	0.15191E-03
147.60	0.39603E-04	0.24189E-03	0.15297E-03
