# Supplementary material for: Modeling Fatty Acid Transfer from Artery to Cardiomyocyte
Source: PLoS Comput Biol. 2015 Dec 16;11(12):e1004666. doi: 10.1371/journal.pcbi.1004666 (PMC4682637; doi:10.1371/journal.pcbi.1004666)
Supplement: S1 Read — (PDF) [file pcbi.1004666.s002.pdf]

Program designed by:
Theo Arts
Maastricht University
Department of Biomedical Engineering
Email: t.arts@maastrichtuniversity.nl
Date: Oct 14, 2015

```
PName = "D:\\TheoArts\\Manuscripten\\GerJimArt\\K220JimData\\FA\\FAdata\\30056FA\\";
FName = "3005612.tac";
{CoFa, CoAlb} = {0.1, 0.11} mmol / l;
VPc = 402 mLPg;
```

Reading lines of 'Data' according to used format of *.tac files
Data = raw data, read from *.tac-file
ReadString reads data string from Data[[i]]
ReadNum reads data number from Data[[i]]
ReadRow[j] reads 1 line of data matrix from Data[[j]]

```
ReadDataFile[FName_, PName_] :=
 Module[{Data, i, iDat, iDatNext, nRow, nCol, ColNames, ReadString, ReadNum, ReadRow},
  Data = Import[StringJoin[PName, FName]];

  ReadString := Module[{d, pos, PosB, PosE},
    d = StringJoin[ Data[[i]] ]; (* remove commas *)
    pos = StringPosition[d, "'"];
    PosB = pos[[3, 1]] + 1;
    PosE = pos[[4, 1]] - 1;
    StringTake[d, {PosB, PosE}]
   ];

  ReadNum := Module[{d, pos},
    d = StringJoin[ Data[[i]] ]; (* remove commas *)
    pos = StringPosition[d, "'"];
    ToExpression[StringTake[d, {pos[[2, 1]] + 1, StringLength[d]}]]
   ];

  ReadRow[j_] := Module[{d, str, row},
    d = Data[[j, 1]];
    str = StringToStream[d];
    row = Read[str, {Number, Number, Number, Number}];
    Close[str];
    row
   ];

  i = 1; TacFileName = ReadString;
  i = 4; ExpName = ReadString;
  i = 5; iDat = i; iDatNext = ReadNum + iDat + 1;
  i = iDat + 2; HeartMass = ToExpression[ReadString] * g;
  i = iDat + 3; LvRvMass = ToExpression[ReadString] * g;

  i = iDatNext;  iDat = i; iDatNext = ReadNum + iDat + 1;
  i = iDatNext;  iDat = i; iDatNext = ReadNum + iDat + 1;

  i = iDat + 2;  CoPalmitateAlbumin = ReadString;
```

```
  i = iDat + 3; Flow = ToExpression[ReadString] * ml min^-1;
  i = iDat + 4; Pressure = ToExpression[ReadString] * mmHg;
  i = iDat + 11; HeartRate = ToExpression[ReadString] * min^-1;
  i = iDat + 12; VInject = ToExpression[ReadString] * ml;
  i = iDat + 13; TInject = ToExpression[ReadString] * s;
  i = iDat + 18; RefScale = ToExpression[ReadString];
  i = iDat + 19; DiffScale1 = ToExpression[ReadString];
  i = iDat + 20; DiffScale2 = ToExpression[ReadString];
  i = iDat + 23; TSample1 = ToExpression[ReadString] * s;
  i = iDat + 24; TSample2 = ToExpression[ReadString] * s;

  i = iDatNext; iDat = i; iDatNext = ReadNum + iDat + 1;
  i = iDatNext; iDat = i; iDatNext = ReadNum + iDat + 1;
  i = iDatNext; iDat = i; iDatNext = ReadNum + iDat + 1;
  i = iDat + 2; nRow = ReadNum;
  i = iDat + 3; nCol = ReadNum;
  i = iDat + 4; ColNames = StringJoin[Data[[i]] ];
  iDat = i;
  Mat = Table[ReadRow[j], {j, iDat + 1, iDat + nRow}];
  {Time, AlbB, SucB, FaB} = Transpose[Mat]; (* collected samples in bins *)
 ]

ReadDataFile[FName, PName]
```
