# Supplementary material for: Modeling Fatty Acid Transfer from Artery to Cardiomyocyte
Source: PLoS Comput Biol. 2015 Dec 16;11(12):e1004666. doi: 10.1371/journal.pcbi.1004666 (PMC4682637; doi:10.1371/journal.pcbi.1004666)

Program designed by:
Theo Arts
Maastricht University
Department of Biomedical Engineering
Email: t.arts@maastrichtuniversity.nl
Date: Oct 14, 2015

```
In[7]:= (*SetDirectory["C:\\Users\\TheoArts\\Manuscripten\\GerJimArt\\ModelMathematica\\"];*)
```

Measurements

Local function library:
RangeDx[XMin_, XMax_, DX_]
NConvolve[f_, g_, Rgf_, Rgg_, dx_]
NLogConvolve[f_, g_, Rgf_, Rgg_, dLnx_]
NDeconvolution[F_, G_, xMinMaxf_, xMinMaxg_, Dx_, W2ndDer_]
FunctionDotProduct[hx_, fxy_, Rgxdx_, Rgydy_]
NInverseFunction[Fu_, Rgxdx_]
NIntegrateFunction[Fu_, Rgxdx_]
ReInterpolation[ft, tMinMaxDt]
ReInterpolation2D[fxt_, xMinMaxDx_, tMinMaxDt_]
NFindZero[f_, LoHiTol_]
FitPar[p_, Fu_,TolNmax_]
Hanning[x]

```
In[8]:= RangeDx[XMin_, XMax_, DX_] := Module[{n},
   n = Round[(XMax - XMin) / DX];
   {XMin + (XMax - XMin) Range[0, n] / n, (XMax - XMin) / n}
  ]
```

```
In[9]:= NConvolve[f_, g_, Rgf_, Rgg_, dx_] := Module[{nf, ng, Listf, Listg, F, G, H, nh, X},
    (* Numerical convolution of functions f(x) and g(x),
    defined within intervals (Lo<x<Hi): Rgf={Lo,Hi} and Rgg={Lo,Hi},
    respectively. Outside these intervals,
    functions f and g are considered zero. dx=Discritization distance *)
    nf = Round[(Rgf[[2]] - Rgf[[1]]) / dx];
    ng = Round[(Rgg[[2]] - Rgg[[1]]) / dx];
    Listf = Table[Rgf[[1]] + i dx, {i, 0.5, nf}]; (* sampled x of fu f(x) *)
    Listg = Table[Rgg[[1]] + i dx, {i, 0.5, ng}]; (* sampled x of fu g(x) *)
    F = Map[f, Listf]; G = Map[g, Listg];
    H = ListConvolve[F, G, {1, -1}, 0] dx;
    nh = Length[H];
    X = Table[Rgf[[1]] + Rgg[[1]] + i dx, {i, 0, nh + 1}];
    Interpolation[ Transpose[{X, Join[{0}, H, {0}] }] ]
   ];
```

In[10]:=
```
(*Clear[F,G,RgfLn,RggLn,H,eps];*)
NLogConvolve[f_, g_, Rgf_, Rgg_, dLnx_] := Module[{F1, G1, RgfLn, RggLn, H1, eps},
  Clear[F1, G1, RgfLn, RggLn, H1, eps];
  F1 = Function[ f[Exp[#]] Exp[#] ]; (* Kernel *)
  G1 = Function[ g[Exp[#]] ]; (* function of time *)
  eps = 10^-4;
  RgfLn = Log[Clip[Rgf, {eps, Infinity}]];
  RggLn = Log[Clip[Rgg, {eps, Infinity}]];
  H1 = NConvolve[F1, G1, RgfLn, RggLn, dLnx];
  Function[H1[Log[#]]]
 ]
```

FunctionDotProduct[hx_, fxy_, Rgxdx_, Rgydy_]:
function h of [x], function fxy of [x,y], Rgxdx={Lo,Hi,dx] so that Lo<x<Hi, Rgydy like Rgxdx
result: if h[x], fxy[x,y] were vector and matrix, result[y]==h[x] . fxy[xy]

In[11]:=
```
FunctionDotProduct[hx_, fxy_, xMinMaxDx_, yMinMaxDy_] := Module[{xMin, xMax, dx, nx,
    yMin, yMax, dy, ny, Hx, Gy, Fxy, X, Y},
   {xMin, xMax, dx} = xMinMaxDx;
   {yMin, yMax, dy} = yMinMaxDy;
   nx = Round[(xMax - xMin) / dx]; X = xMin + (xMax - xMin) Range[0, nx] / nx;
   ny = Round[(yMax - yMin) / dy]; Y = yMin + (yMax - yMin) Range[0, ny] / ny;
   Hx = Table[hx[x], {x, X}] dx;
   Fxy = Table[fxy[x, y], {x, X}, {y, Y}];
   Gy = Hx.Fxy;
   Interpolation[Transpose[{Y, Gy}]]
  ];
```

Hanning[x]=Cos[Pi x/2]^2 for Abs[x]<1, else =0

In[12]:=
```
Hanning = Cos[Pi / 2 Clip[#, {-1, 1}]]^2 &;
```

Numerical deconvolution by minimizing the 2nd derivative. F=kernel function, G=large range function, xMin-Maxf,g=ranges of function, Dx=discretization interval, W2ndDer= factor to restrict the 2nd derivative (e.g. value=0.05).
{bb,aa} program lines cause the tail to be smoothed more than the peak

```
In[13]:= NDeconvolution[F_, G_, xMinMaxf_, xMinMaxg_, Dx_, W2ndDer_] :=
  Module[{xfMin, xfMax, xgMin, xgMax, xg, dx, xf, fxT, gxT, ng, nf, ffT, B1, A, ConvT, xT, bb, aa},
   {xfMin, xfMax} = xMinMaxf;
   {xgMin, xgMax} = xMinMaxg;
   {xg, dx} = RangeDx[xgMin, xgMax, Dx];
   xf = Range[xfMin, xfMax, Dx];
   fxT = Table[F[x], {x, xf}]; (* sampled functions F[x] *)
   gxT = Table[G[x], {x, xg}]; (* sampled functions G[x] *)
   ng = Length[xg];
   nf = Length[xf];
   ffT = ListConvolve[{fxT}, IdentityMatrix[ng], 1, 0];
   bb = Accumulate[Abs[gxT]];
   aa = DiagonalMatrix[ 5 bb + 0.2 Mean[bb] ];
   B1 = ListConvolve[{{+1}, {-1}}, W2ndDer aa, 2, 0] / dx;
   (*B1=ListConvolve[{{+1},{-1}},W2ndDer IdentityMatrix[ng],2,0]/dx;*)
   A = ffT.Transpose[ffT] + B1.Transpose[B1];
   ConvT = gxT.Transpose[ffT].PseudoInverse[A];
   xT = xfMin + xgMin + (Range[ng] - 1) dx;
   Interpolation[Transpose[{xT, ConvT}]]
  ]
```

NInverseFunction[Fu,Rgxdx]: Fu=function of x, Rgxdx={Lo,Hi,dx} with Lo<x<Hi, dx=numerical sampling interval
Result: function Result so that NInverseFunction[Fu,{Lo,Hi,dx}][Fu[x]]==x

```
In[14]:= NInverseFunction[Fu_, Rgxdx_] := Module[{Lo, Hi, dx, dx1, X, FuX, n},
    {Lo, Hi, dx} = Rgxdx;
    n = Round[(Hi - Lo) / dx];
    dx1 = (Hi - Lo) / n;
    X = Lo + Range[0, n] * dx1;
    FuX = Map[Fu, X];
    Interpolation[Transpose[{FuX, X}]]
   ];
```

NIntegrateFunction[Fu_,Rgxdx] renders Integral[x] from lower boundary Rgxdx[[1]]. Function defined between lower and upper x-boundary
Rgxdx={lower x-boundary, upper x-boundary, numerical increment}

```
In[15]:= NIntegrateFunction[Fu_, Rgxdx_] := Module[{Lo, Hi, dx, dx1, X, FuX, XInt, IntFuX},
    {Lo, Hi, dx} = Rgxdx;
    n = Round[(Hi - Lo) / dx];
    dx1 = (Hi - Lo) / n;
    X = Range[0.5, n] dx1;
    FuX = Map[Fu, X] dx1;
    XInt = Lo + Range[0, n] dx1;
    IntFuX = Join[{0}, Accumulate[FuX]];
    Interpolation[Transpose[{XInt, IntFuX}]]
   ];
```

ReInterpolation[ft,{tMin,tMax,dt}] reinterpolates and removes history

In[16]:=
```
ReInterpolation[ft_, tMinMaxDt_] := Module[{tMin, tMax, dt, n, tT, ftT},
   {tMin, tMax, dt} = tMinMaxDt;
   {tT, dt} = RangeDx[tMin, tMax, dt];
   ftT = Table[{t, ft[t]}, {t, tT}];
   Interpolation[ftT]
  ];
```

In[17]:=
```
ReInterpolation2D[fxt_, xMinMaxDx_, tMinMaxDt_] :=
  Module[{xMin, xMax, dx, tMin, tMax, dt, xT, tT, fxtT},
   {xMin, xMax, dx} = xMinMaxDx;
   {tMin, tMax, dt} = tMinMaxDt;
   {xT, dx} = RangeDx[xMin, xMax, dx];
   {tT, dt} = RangeDx[tMin, tMax, dt];
   fxtT = Flatten[Table[{{x, t}, fxt[x, t]}, {x, xT}, {t, tT}], 1];
   Interpolation[fxtT]
  ];
```

FitPar[p, Fu, TolNmax] find best parameter fit. p=start value vector of parameters, Fu[p]= function of parameter vector, rendering error vector, which should be minimized. TolNmax= {relative error in p values, maximum number of iterations}

In[18]:=
```
f[nA_] := {nA[[1]] + 1, (1 - 1 / (nA[[1]] + 2)) Sqrt[ 1 - nA[[2]]^2 ]};
pMat[nMax_] := Module[{},
   {n, A} = {0, 0}; p = {{0}};
   While[n < nMax,
    aux = f[{n, A}];
    {n, A} = aux;
    a21 = Table[-A / n, {n}];
    a12 = Append[Table[0, {n}], A];
    a1 = Append[p, a21];
    p = Join[a1, Transpose[{a12}], 2];
   ];
   Transpose[p[[2 ;;]]]
  ];
```

```
In[20]:= FitPar[p_, Fu_, TolNmax_] := Module[{dxMax, IM, P, XMean2, dx2,
      nX, np, x, dx, X, XMean, iT, Tol, Y, nY, YMean, DX, DY, DXt, DYt, Aux, Aux2},
    {Tol, iT} = TolNmax;
    dxMax = 10 Tol;
    np = Length[p];
    x = Log[p];
    P = pMat[np];
    X = Table[x, {np + 1}] + dxMax P;
    dx = Table[0, {np}];
    nX = Length[X];
While[iT > 0 ,
     XMean = Mean[X];
     Y = Map[Fu, Exp[X]];
     nY = Dimensions[Y][[1]];
     YMean = Mean[Y];
     DX = X - Table[XMean, {nX}];
     DY = Y - Table[YMean, {nY}];
     DXt = Transpose[DX];
     DYt = Transpose[DY];
     dx = -YMean.PseudoInverse[DYt.DY].DYt.DX; (* deviation from current xMean *)
     dx *= Min[1, dxMax Sqrt[np] / (Norm[dx] + Tol)];
     (*dx2=dx+XMean-XMean2; (* deviation from previous xMean = xMean2 *)
     dx2*=Min[ Norm[dx2],dxMax Sqrt[ nP]  ] / Norm[dx2];*)
     (* maximalization of step size *)
     X += Table[dx, {nX}];
     iT = If[Norm[dx] < Tol, Floor[0.3 iT], iT - 1];
     Print[{iT, Exp[XMean], Plus @@ Flatten[Y^2]}];
    ];
    XMean = Mean[X];
    Exp[XMean]
   ];
```

NFindZero[f,{Lo,Hi,Tol}] finds zero with starting values Lo and Hi by linear interpolation. Tol=relative tolerance relative to Abs[f[Lo]-f[Hi]]

```
In[21]:= NFindZero[f_, LoHiTol_] := Module[{Lo, Hi, Tol, fLo, fHi, x1, x2, x3, f1, f2, nit},
   {Lo, Hi, Tol} = LoHiTol;
   x1 = Lo; x2 = Hi;
   f1 = f[x1]; f2 = f[x2]; fLo = f1; fHi = f2;
   nit = 10; (* max number of iterations *)
   While[ nit > 0 && Abs[(f1 - f2) / (fLo - fHi)] > Tol,
    nit--;
    x3 = (f1 x2 - f2 x1) / (f1 - f2);
    x1 = x2; f1 = f2;
    x2 = x3; f2 = f[x2];
   ];
   x2
  ]
```

SI units are defined. Unit conversions

```
In[22]:= FAC = 1.2;
```

In[23]:=
```
Clear[mLPminPg];
nano = 10^-9;
μ = 10^-6;
milli = 10^-3;
l = 10^-3 m^3;
ml = milli l;
cm = 0.01 m;
μm = μ m;
nm = nano m;
min = 60 s;
g = 0.001 kg;
mmol = 0.001 mol;
Dim0 = {mol → 1, m → 1, s → 1, kg → 1}; (* setting equations dimensionless *)
```

Measurements LM and EM microscopy
V=volume fraction, S=membrane Area/Volume (m^2/m^3, dDiff= effective diffusion layer thickness

In[36]:=
```
VCap = 0.094; (*Capillaries [-] *)
VEc = 0.018;(*Endothelial cells [-] *)
VIs1 = 0.019;(*Interstitium 1 [-] *)
VIs2 = 0.060;(*Interstitium 3 [-] *)
VMy = 0.731;(*Myocytes [-] *)
VTt = 0.010;(*T-tubuli [-] *)
VBv = 0.059;(*Blood vessels, underestimation [-] *)
VIsCell = 0.010; (*Interstitial cells [-] *)
SCap = 75000 / m /. Dim0; (*Area/Volume Cap-Ec [1/m] *)
SEc = 82000 / m /. Dim0; (*Area/Volume Ec-Is1[1/m] *)
SIs1 = 89000 / m /. Dim0; (*Area/Volume Is1-Myo[1/m] *)
SIs2 = 94000 / m /. Dim0; (*Area/Volume [1/m] *)
dDiffEc = 187 nm /. Dim0; (*Diffusion thickness [m] *)
dDiffIs1 = 160 nm /. Dim0; (*Diffusion thickness [m] *)
dMembrane = 5.0 nm /. Dim0;(*Musters*)
dCap = 5.2 μm /. Dim0; (* Capillary diameter *)
PartitionCoef = 8 × 10^5; (* palmitate, water-membrane, Kleinfeld *)
```

Physical properties (Basic data)

```
In[53]:= DiffAlbumin = 9.35 × 10^-11 ;(* [m2/s] Weisiger*)
DiffOleate = 5.0 × 10^-10 ;(*[ m2/s] Weisiger*)
MolWeightAlbumin = 67000;(* Wiki/NIH *)
MolWeightCpMyo = 15000;(* Ger/Check *)
MolWeightPalmitate = 256;
MolWeightOleate = 282;
Density = 1050;

DiffPalmitate = N[Sqrt[MolWeightOleate / MolWeightPalmitate] DiffOleate];
DiffFa = DiffPalmitate; (* Free palmitate in water *)

SCapEc = SCap;
SEcIs = SEc;
SIsMyo = SIs1;
VFaMemCapEc = PartitionCoef SCapEc dMembrane;
VFaMemEcIs = PartitionCoef SEcIs dMembrane;
VFaMemIsMyo = PartitionCoef SIsMyo dMembrane;
(* [mol m-3] Fa-storage space of single Ec membrane around capillary *)
```

Capillary (Cap) compartment

```
In[68]:= kCap = 8.47 × 10^-6;  (* [mol/m3], equilibrium constant Cp+Fa<->Cp+Fa *)
TaukCap = 0.083 ; (*Albumin, Richieri*)
TaukAlb = 0.007 ; (*Albumin oleate, Bojesen*)
TaukCap = TaukCap;
NCp = 3; (* number of Fa binding sites Cp on Alb molecule *)
DiffCpCap = DiffAlbumin;
CoCpTAr = CoAlb NCp /. Dim0 ; (* Arterial Cp-concentration *)
CoCpTCap = CoCpTAr;
(* diameter Albumin 8.5-11 nm, Kizelev MA, Biofizika, 2001 May-Jun;
46(3):423-7. *)
dCpCap = 5.0 nm /. Dim0;
(* Fa-release by direct contact of albumin to membrane *)
dDiffCap = 0.15 dCap; (* estimate length of cross-diffusion pathway*)
```

Endothelial (Ec) compartment

```
In[78]:= kEc =  kCap;
TaukEc = TaukAlb; (*?? same as kCap release ?? +++ *)
MolWeightCpEc = MolWeightCpMyo;
DiffCpEc = DiffAlbumin Sqrt[MolWeightAlbumin / MolWeightCpEc]; (* low molecular weight *)
CoCpTEc = 0.005 (CoCpTAr / 0.33) ^0.3; (* low concentration, varies slightly *)
CoCpTEc = 0.007;  (* low, but fixed concentration *)
dCpEc = dCpCap (MolWeightAlbumin / MolWeightCpEc) ^ (-1 / 3);
(* Fa-release by direct contact of albumin to membrane *)
```

Peri-capillary interstitium (Is) compartment

```
In[84]:= kIs = kCap;
TaukIs = TaukAlb ;
DiffCpIs = DiffCpCap; (* low molecular weight *)
CoCpTIs = 0.86 CoCpTAr; (* Albumin, equilibrium with Cap +++ *)
dCpIs = dCpCap;(* Fa-release by direct contact of albumin to membrane *)
VIs = VIs1;
dDiffIs = dDiffIs1;
```

Myocyte (Myo) compartment

```
In[91]:= kMyo = kCap; (*++++*)
TaukMyo = TaukAlb ; (*++++*)
DiffCpMyo = DiffAlbumin Sqrt[MolWeightAlbumin / MolWeightCpMyo]; (* low molecular weight *)
CoCpTMyo = 0.17;(*++ Vork et al*)
dCpMyo = dCpCap (MolWeightAlbumin / MolWeightCpMyo) ^ (-1 / 3);
(* Fa-release by direct contact of albumin to membrane *)
dDiffMyo = 2.5 × 10^-6; (* Estimate of diffusion path length *)
```

Measured data

```
In[97]:= qTissue = Flow Density (kg / m ^3) / LvRvMass s ;
Print["File name: ", FName]
Print["LvRvMass= ", LvRvMass];
Print["Flow/Tissue Volume [1/s]: ", qTissue ];
Print["Albumin, Palmitate concentration [mol/m^3]: ", {CoAlb, CoFa} m ^3 / mol];
Print["Capillary mean transit time (TauCap) [s]: ", TauCap = VCap / qTissue]

File name: 3005612.tac

LvRvMass= 0.00836 kg

Flow/Tissue Volume [1/s]: 0.0351675

Albumin, Palmitate concentration [mol/m^3]: {0.11, 0.1}

Capillary mean transit time (TauCap) [s]: 2.67293
```

UnSample[f] corrects for smoothing due to relatively large collection time per sample by amplifivcation of HF fluctuations
SubSample[f] doubles sampling frequency, while introducing some LF-filtering
Measured dilution curve
AlbB, SucB, FaB are originally sampled concentrations
T1, Alb and Fa represent measured samples of time, [Albumin] and [Fatty acid=palmitate]

Functions
UnSample[f_]
SubSample[f_]
Sample2Fu[Tracer_]

Converts measured samples {Time, AlbB, FaB} to multiple samples, ready for function interpolation {T1, Alb, Fa}

```
In[106]:= iSub = 3;
T1 = Nest[SubSample, Time, iSub];
Alb = Nest[SubSample, UnSample[AlbB], iSub];
Fa = Nest[SubSample, UnSample[FaB], iSub];
```

Total Carrier Protein (albumin) binding site [Cp] and [Fa] concentrations as fu[t], CpT and FaT represent total concentrations in experimental samples

```
In[110]:= CpFt1 = Sample2Fu[Alb];
FaFt1 = Sample2Fu[Fa];
CpT1 = Transpose[{T1, Alb}];
FaT1 = Transpose[{T1, Fa}];
tMax1 = Max[T1];
```

Graphical comparison functions obtained from discrete samples
Amplitude calibration (1/ICp) and time shift (TShift) so that integral of Cp[t]==1 and location of peak is appropriate for further analysis
Ratio of [Fa] and [Cp] is preserved

```
In[115]:= dt = 0.5; t = Range[0, tMax1, dt]; (* Equidistant resampling *)
ICp = Plus @@ CpFt1[t] dt + CpFt1[tMax1] / tMax1;
(* added integration of early function part and tail *)
ICpP = Plus @@ Clip[ CpFt1[t] - 0.5 Max[ CpT1[[All, 2]] ], {0.0, Infinity}] dt;
(* added integration of early function part and tail *)
IFa = Plus @@ FaFt1[t] dt + FaFt1[tMax1] / tMax1;
(* added integration of early function part and tail *)
(* added integration of early function part and tail *)
ItCp = Plus @@ (t CpFt1[t]) dt; (* mean transit time *)
ItCpP = Plus @@ (t Clip[CpFt1[t] - 0.5 Max[CpT1[[All, 2]]], {0, Infinity}]) dt;
(* mean transit time *)
Clear[t];
TShift = + ItCpP / ICpP - 3.0 TauCap;
FeedThrough = IFa / ICp; FAux = Clip[FeedThrough, {0.001, 0.999}]; (*Avoid negative uptake*)
ExtractionFa = 1 - FAux;

CpFt = CpFt1[# + TShift] / ICp &;
FaFt = FaFt1[# + TShift] (FAux / FeedThrough) / ICp &;
T = T1 - TShift;
CpT = Transpose[{T, Alb / ICp}]; (* similar time shift of measured data *)
FaT = Transpose[{T, (FAux / FeedThrough) Fa / ICp}];
(* similar time shift of measured data *)
tMax = Max[T];
```

ImS= sample points [Alb] and [FaT]; Log=logarithmic schaling
Ima= Albumin Washout curves CpFt[t] with Integral CpFt[t] dt=1 by amplitude scaling.
Fatty acid Washout curve FaFt[t] with same amplitude scaling.
Shift in time by 3.0 TauCap relative to peak of curve.
Tail of curve follows Constant t^-2, being slow washout near the walls of injection chamber.
Graphical comparison of data points and generated functions

In[131]:=
```
Tm = 15;
TS = Time - TShift;
CpS = Transpose[{TS, AlbB}]; FaS = Transpose[{TS, FaB}]; (* measured samples *)
Ima = Plot[{CpFt[t], FaFt[t]}, {t, 0, Tm}, PlotRange → {{0, Tm}, All}];
ImS =
  ListPlot[{CpS, FaS}, PlotRange → {{0, Tm}, All}, PlotStyle → Directive[PointSize[Medium]] ];
ImLoga = LogPlot[{CpFt[t], FaFt[t]}, {t, 0, tMax}, PlotRange → {All, {10^-6, 1}}];
ImLogS = ListLogPlot[{CpS, FaS},
   PlotRange → {All, {10^-6, 1}}, PlotStyle → Directive[PointSize[Medium]] ];
Show[Ima, ImS]
Show[{ImLoga, ImLogS}]
Print["Total Cp, Total Fa, Extraction Fa: ", {ICp, IFa, ExtractionFa}];
```

Out[138]=
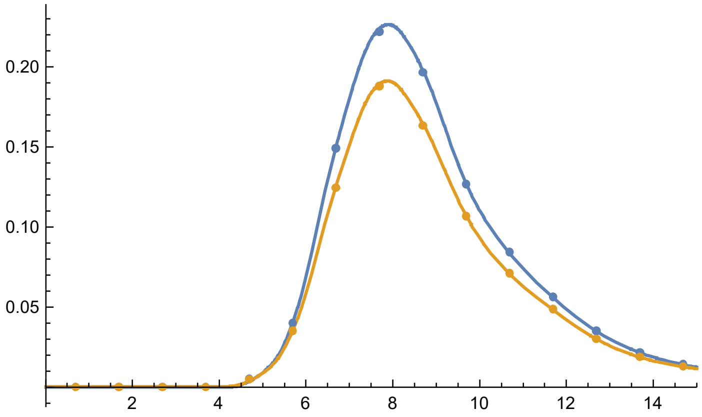

Out[139]=
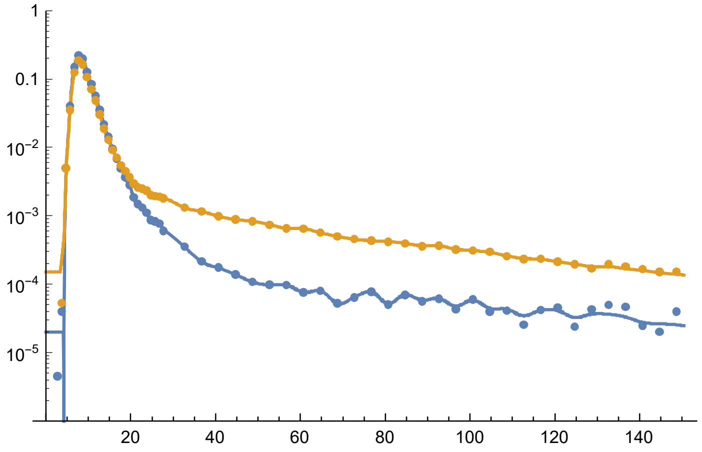


```
Total Cp, Total Fa, Extraction Fa: {1.00002, 0.913244, 0.0867777}
```

Albumin in Large blood vessels and Capillaries

Capillary length dispersion model
Capillary velocity from A→V decays inversely proportional with distance from capillary entrance.
Mean transit time= capillary volume/flow
x=normalized capillary length.
Normalized Mean transit time = 1
Ax[x]= normalized capillary cross-section [x], Ax[0]=1, Integral(x:0→1) A[x]dx=1, also used as tissue weight function
Qx[x]= flow fraction [x], Q[0]=1
hx[x]= flow leakage fraction= -Qx'[x]
ux[x]= normalized mean velocity[x] =Qx[x]/Ax[x], u[0]=1
Taux[x]= delay[x] with time normalized to TauCap

In[141]:=
```
TauCap *= 1;(*+++FLOW CHANGE+++*)
Width = 0.5;
eps = 0.0001;
Ax = (1 - Erf[Width / 4 + Log[eps + #] / Width]) / 2 &;
Qx = (1 - Erf[3 Width / 4 + Log[eps + #] / Width]) / 2 &;
hx = -Qx'[Clip[#, {eps, Infinity}]] &;
ux = (x1 = Clip[#, {eps, Infinity}]; Qx[x1] / Ax[x1]) &;
dx = 0.05; (* sampling distance *)
xMax = 3.0; (* xMax for practical use *)
Taux = NIntegrateFunction[1 / (ux[#]) &, {0, xMax, dx}];
Print["Dispersion Width capillary length= ", Width];
SMem = NIntegrateFunction[Ax[#] &, {0, xMax, dx}];
```

```
Dispersion Width capillary length= 0.5
```

```
Plot[{hx[x], ux[x], Qx[x], Ax[x], Taux[x], SMem[x]}, {x, 0, xMax}, PlotRange → {All, {0, 2}}]
```

Out[153]=
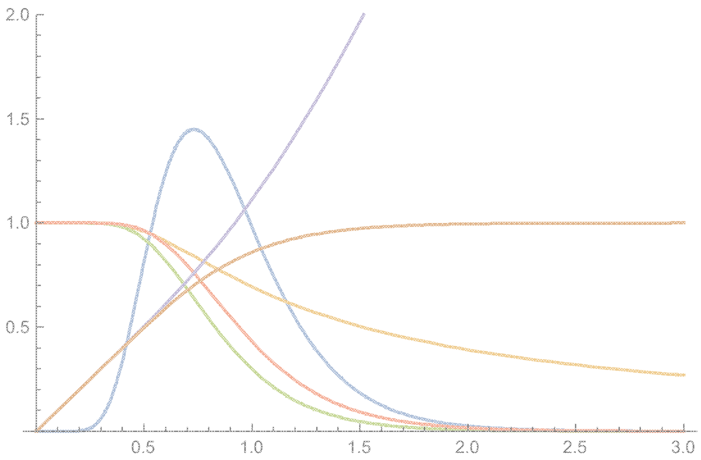

Concentration CCpTArt[t] at arterial side of capillary is found by deconvolution with impulse response ht[t]. Convolution of CCpTArt[t] with hT[t] resembles CpFt[t]. Graph of peak shows comparison of reconstructed Albumin curve with dots representing measurements. The earlier peak represents CCpTArt[t] as found by deconvolution. 2nd graph shows logarithm of concentration CCpTArt[t].
t=normalized time=T/TauCap

In[154]:=
```
Tau1 = 0.1 * TauCap; (* width of input pulse, should be << width of dilution output peak *)
nTau1 = 3;
D2nd = 0.01;
ft = (# / Tau1) Exp[- (# / Tau1 - nTau1) ^2] &;
aux = NIntegrateFunction[ft, {0, tMax, dt}][tMax];
CCpTt = ft[#] / aux &; (* Artificial Unit integral pulse function, used as input *)
CpTCapxt0[x_, t_] := CCpTt[t - Taux[x] TauCap] Exp[-5 x];
(* Splitting for more accurate solution *)
CpTCapxt[x_, t_] := CpTCapxt0[x, t] + dCpTCapxt[x, t];
Eq1 = D[CpTCapxt[x, t], t] +
     (ux[x] / TauCap) ( D[CpTCapxt[x, t], x] - D2nd D[CpTCapxt[x, t], x, x]) == 0.0;
Bc1 = {dCpTCapxt[0, t] == 0,
    dCpTCapxt[x, 0] == 0,
    dCpTCapxt[xMax, t] == 0};
tm = Min[10 TauCap + 2 Tau1, tMax];
dt = 0.2; (* time step for discretization *)
SolB = Last[NDSolve[{Eq1, Bc1}, {dCpTCapxt}, {x, 0, xMax}, {t, 0, tm}, MaxStepSize → 0.05]];
tm -= nTau1 Tau1;

ht1 = FunctionDotProduct[hx, CpTCapxt[#1, #2 + nTau1 Tau1] /. SolB &, {0, xMax, dx}, {0, tm, dt}];
a = NIntegrateFunction[ht1[#] &, {0, tm, dt}][tm];
ht = ht1[#] / a &; (* transcapillary impulse response function *)

Plot[{0.1 CCpTt[t], ht[t]}, {t, 0, 0.5 tm}, PlotRange → All]
(*Plot[{Log[CCpTt[t]],Log[ht[t]]},{t,0,tm},PlotRange→{-10,2}]*)
```

Out[171]=

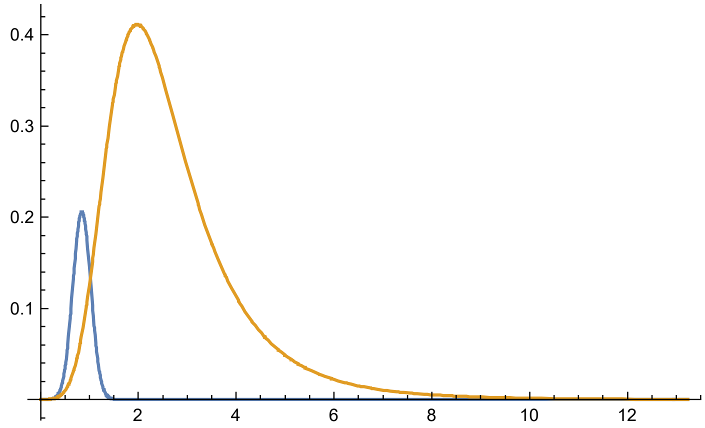

CCpTArt[t]=arterial total Cp concentration, found by deconvolution, and normalized to unit integral. CCpTVet[t]= related venous concentration, found by convolution. Should be (and appears) the same as when using the complete model of Ct-dilution

In[172]:=
```
C2nd = 0.15; (* +++ 2nd derivative effect to cut off ocillations in deconvolution *)
CCpTArt1 = NDeconvolution[ht, CpFt, {0, tm}, {0, tMax}, dt, C2nd];
f2 = ReInterpolation[Clip[CCpTArt1[#], {10^-5, Infinity}] &, {0, tMax, dt}];

i = 0;
While[i < 2,
  i++;
  g3 = NConvolve[ht, f2, {0, tm}, {0, tMax}, dt];
  dg3 = CpFt[#] - g3[#] &;
  df3 = NDeconvolution[ht, dg3, {0, tm}, {0, tMax}, dt, 2^(-i) C2nd];
  f2 = ReInterpolation[Clip[f2[#] + df3[#], {10^-5, Infinity}] &, {0, tMax, dt}];
 ];

CCpTArt2 = f2;
Aux = NIntegrateFunction[CCpTArt2, {0, tMax, dt}][tMax];
CCpTArt = ReInterpolation[(CCpTArt2[#] / Aux) &, {0, tMax, dt}];
CCpTVet = NConvolve[ht, CCpTArt, {0, tm}, {0, tMax}, dt];
```

CCpTArt[t]=Arterial input concentration found by deconvolution. CCpTVet[t]= venous output by convolution. Red dots: experimental data samples

```
In[181]:= Show[Plot[{CCpTArt[t], CCpTVet[t]}, {t, 0, tm}, PlotRange → {{0, tm}, All}],
  ListPlot[CpS, PlotStyle → Directive[PointSize[Medium], Red]], PlotRange → {{0, 20}, All}]
Show[LogPlot[{CCpTArt[t], CpFt[t], CCpTVet[t]}, {t, 0, tMax}, PlotRange → {10^-6, 1.0}],
  ListLogPlot[CpS, PlotStyle → Directive[PointSize[Medium], Red], PlotRange → {10^-6, 1.0}]]
```

Out[181]=

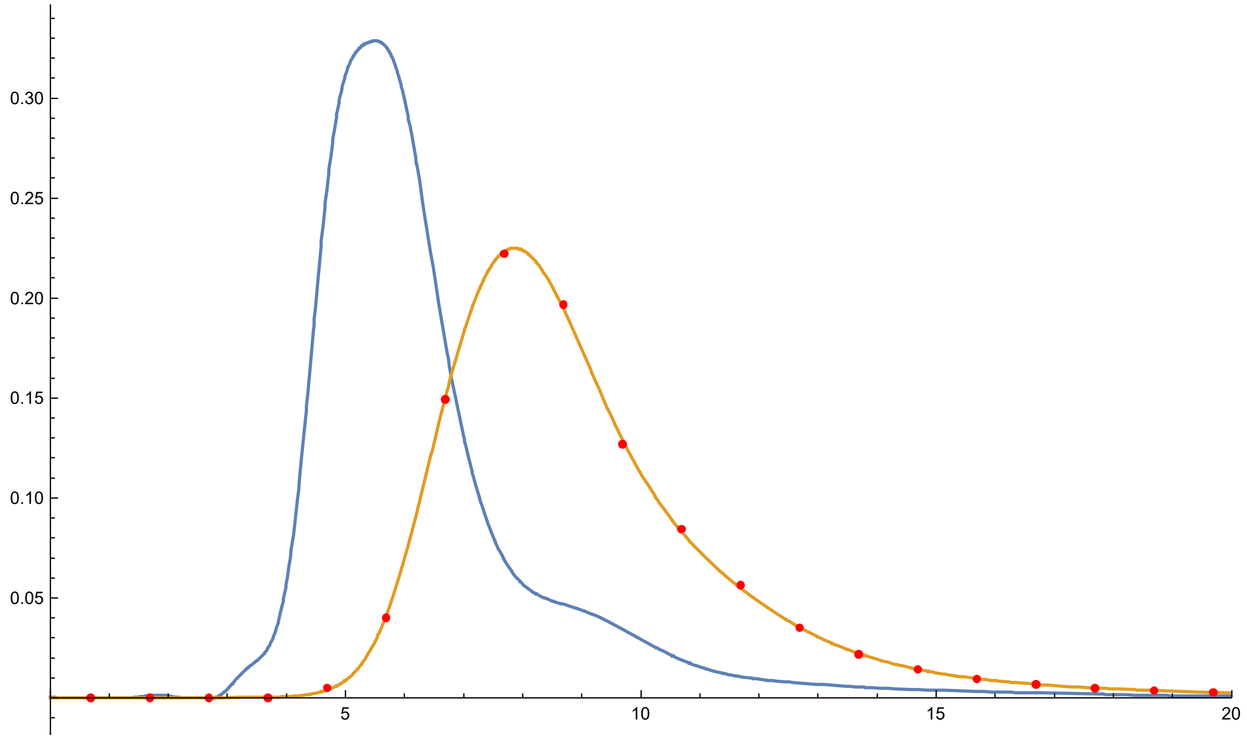

Out[182]=

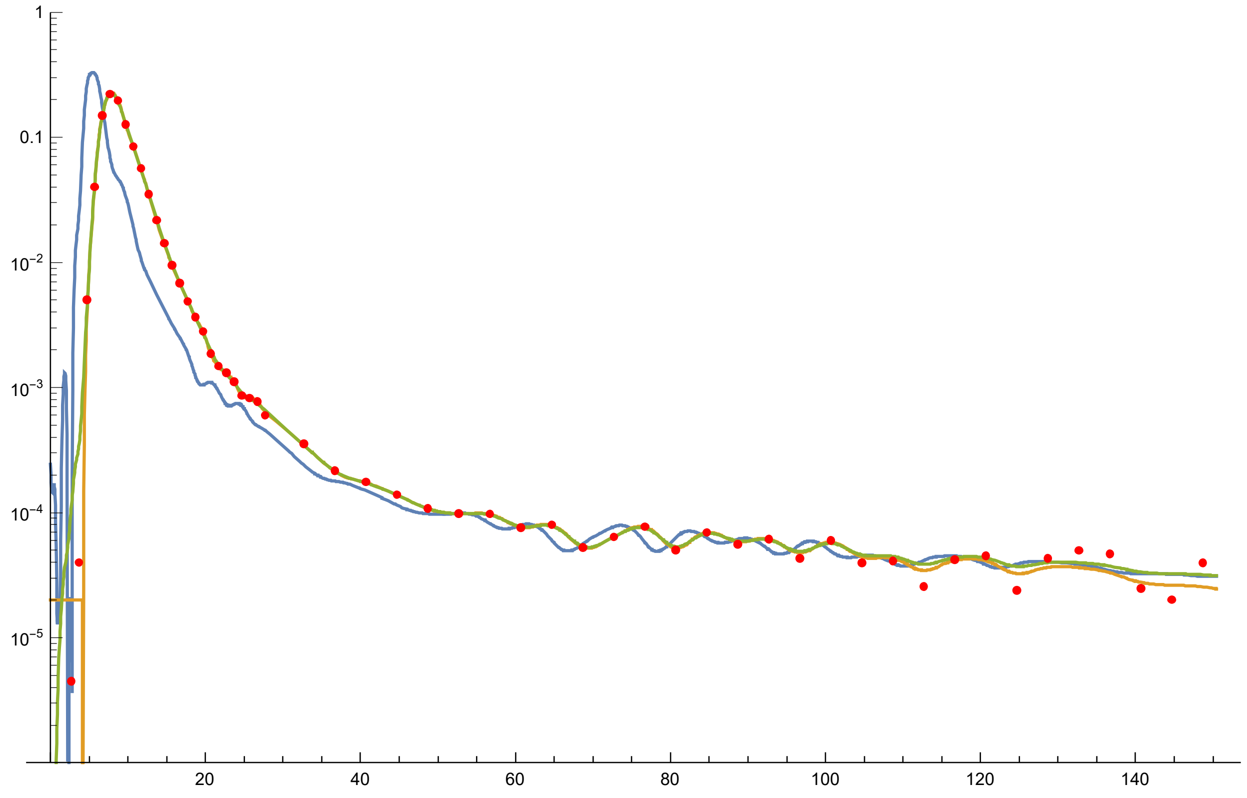

Capillaries Albumin-Fatty Acid

In[183]:=
```
(* steady state total albumin (CpT) concentration *)
CoCpTNorm = 1.5; (* Physiological plasma Fa-binding site concentration *)
CoFaTAr = CoFa m^3 / mol; (* Total Fa concentration *)
```

Fak=[Fa]/k; Sat=saturation, Co= steady state concentration, Cp=carrier protein, k=equilibrium constant, Tauk= decay time constant

Function declarations

In[185]:=
```
SatFak = 1 - 1 / (1 + #) &;(* saturation=fu(Fa/k) *)
FakSat = 1 / (1 - #) - 1 &;(* Fa/k=fu(saturation), inverse of SatFak *)
Para = 1 / Plus @@ (1 / #) &;(* parallel, reciprocal sum of reciprocals *)
Perm = Function[{CoCp, k, Tauk, dCp}, (b = Sqrt[CoCp / (DiffFa k Tauk)];
    b DiffFa (1 + b dCp))];
(* Fa membrane interface permeability [m/s],
b= 1/boundary layer thickness, Co=concentration *)
PermBulk = Function[{Fak, DiffCp, CoCpT, k, dBulk},
   (DiffFa + (1 - SatFak[Fak])^2 DiffCp CoCpT / k ) / dBulk];
(* Fa permeability in layer with facilitated diffusion, carrier protein Cp*)
CoCp = Function[{Fak, CoCpT}, CoCpT (1 - SatFak[Fak])]; (* Free Cp-concentration=Fu[Fa/k]]*)
```

CoCpx= module to calculate Co[x] (steady state concentrations)

In[191]:=
```
CoCpx := Module[{},
   (* Initialization Fa/k[x] values*)
   FakAr = FakSat[CoFaTAr / CoCpTCap]; (* Arterial [Fa]/k *)
   FakVeTarget = FakSat[(1 - ExtractionFa) CoFaTAr / CoCpTCap];
   (* extraction derived from dilution curves *)
   FakEcx = FakVeTarget kCap / kEc &; (* Ec: initialization as function *)
   FakIsx = FakVeTarget kCap / kIs &; (* Is: initialization as function *)
   FakMyo = FakVeTarget kCap / kMyo; (* Myo: initialization as constant *)

   (* Iterative solution of (Fa/k)Myo so that Fa-extraction fits*)
   i = 10; (* Maxiumum number of iterations *)
   Error0 = 10.^-5; (* Error to stop iteration *)
   Error = 0.5; (* Initial error (large) *)
   While[Abs[Error] > Error0 && i > 0,
    i -= 1;
    Clear[FakCapx];

    (* CoCp= concentration of free carrier protein [mol/m3]*)
    CoCpCapx = CoCp[FakCapx[#], CoCpTCap] &;
    CoCpEcx = CoCp[FakEcx[#], CoCpTEc] &;
    CoCpIsx = CoCp[FakIsx[#], CoCpTIs] &;
    CoCpMyo = CoCp[FakMyo, CoCpTMyo];
    (* Perm= permeability membrane interface [m/s] *)
    PermCapx = Perm[CoCpCapx[#], kCap, TaukCap, dCpCap] &;
    PermEcx = Perm[CoCpEcx[#], kEc, TaukEc, dCpEc] &;
    PermIsx = Perm[CoCpIsx[#], kIs, TaukIs, dCpIs] &;
    PermMyo = Perm[CoCpMyo, kMyo, TaukMyo, dCpMyo];
```

```
    (* PermBulk= permeability aqueous compartments *)
    PermBulkCapx = PermBulk[FakCapx[#], DiffCpCap, CoCpTCap, kCap, dDiffCap] &;
    PermBulkEcx = PermBulk[FakEcx[#], DiffCpEc, CoCpTEc, kEc, dDiffEc] &;
    PermBulkIsx = PermBulk[FakIsx[#], DiffCpIs, CoCpTIs, kIs, dDiffIs] &;
    PermBulkMyo = PermBulk[FakMyo, DiffCpMyo, CoCpTMyo, kIs, dDiffMyo];
    (* Ps= permeability surface area product [m3/s] by series of Perm's*)
    PsCapEcx = SCapEc Para[{PermBulkCapx[#], PermCapx[#], PermEcx[#], 2 PermBulkEcx[#]}] &;
    PsEcIsx = SEcIs Para[{2 PermBulkEcx[#], PermEcx[#], PermIsx[#], 2 PermBulkIsx[#]}] &;
    PsIsMyox = SIsMyo Para[{2 PermBulkIsx[#], PermIsx[#], PermMyo, 2 PermBulkMyo}] &;
    (* transport flow [mol/s] *)
    fCapMyox = (FakCapx[#] kCap - FakMyo kMyo) Para[{PsCapEcx[#], PsEcIsx[#], PsIsMyox[#]}] &;
    (* steady state differential equation + boundary condition for free [Cp](x) *)
    EqCox = VCap CoCpTCap SatFak'[FakCapx[x]] FakCapx'[x] ux[x] / TauCap + fCapMyox[x] == 0;
    BcCox = FakCapx[0] == FakAr;

    FakMyo0 = FakMyo; (* store previous value FakMyo *)
    Solx = First[NDSolve[{EqCox, BcCox}, FakCapx, {x, 0, xMax}]];
    (* new solution ODE *)

    FakCapx = FunctionInterpolation[FakCapx[x] /. Solx, {x, 0, xMax}];
    fCapMyox = FunctionInterpolation[fCapMyox[x] /. Solx, {x, 0, xMax}];
    CoFaTCapx = Function[x, CoCpTCap SatFak[FakCapx[x]]];
    CoFaTVe = NIntegrateFunction[CoFaTCapx[#] hx[#] &, {0, xMax, dx}][xMax];
    FakVe = FakSat[CoFaTVe / CoCpTCap]; (* should approach target value *)FakEcx =
     FunctionInterpolation[(FakCapx[x] kCap - fCapMyox[x] / PsCapEcx[x]) / kEc, {x, 0, xMax}];
    FakIsx = FunctionInterpolation[(FakEcx[x] kEc - fCapMyox[x] / PsEcIsx[x]) / kIs,
      {x, 0, xMax}];

    Delta = (FakVeTarget - FakVe) (FakAr - FakMyo0) / (FakAr - FakVe);
    FakMyo += Delta; (* new estimate FakMyo *)
    Aux = Clip[FakMyo, {0.001, 1000.0}];
    FakMyo = Aux;
    Error = FakVe - FakVeTarget
   ];
   (* [CoCp]free in all compartments as fu(x), needed to calculate permeability *)
   CoCpCapx = CoCpTCap (1 - SatFak[FakCapx[#]]) &;
   CoCpEcx = CoCpTEc (1 - SatFak[FakEcx[#]]) &;
   CoCpIsx = CoCpTIs (1 - SatFak[FakEcx[#]]) &;
   CoCpMyo = CoCpTMyo (1 - SatFak[FakMyo]);
  ];
```

```
In[192]:= CoCpx; (* execution of finding steady state concentrations *)
Plot[{FakCapx[x] kCap, FakEcx[x] kEc, FakIsx[x] kIs, FakMyo kMyo}, {x, 0, xMax}, PlotRange → All]
(*Plot[{Log10[CoCpCapx[x]],Log10[CoCpEcx[x]],Log10[CoCpIsx[x]],Log10[CoCpMyo]},{x,0,xMax}]
  Print[{FakAr,FakVe,FakMyo}];*)
```

Out[193]=

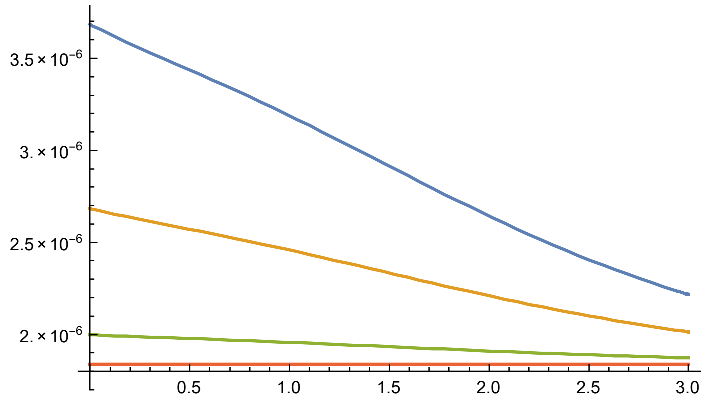

Labeled Fa*[t]-dilution. CFaT*[t]=total [Fa]. Input concentration CFaT*[t] the same as that for CpT*[t].
C indicates 'labeled'

In[194]:=
```
CFaTArt = CCpTArt;
Clear[CFakCapxt, CFaTCapxt0, CFaTCapxt, dCFaTCapxt];
(* Splitting of solution CFakCapxt *)
CFaTCapxt0[x_, t_] := CFaTArt[Clip[t - Taux[x] TauCap, {0, Infinity}]] Exp[-5 x ExtractionFa];
CFaTCapxt[x_, t_] := CFaTCapxt0[x, t] + dCFaTCapxt[x, t];
dCFaTCapxt[x_, t_] := CoCpCapx[x] dCFakCapxt[x, t];
CFakCapxt[x_, t_] := CFaTCapxt[x, t] / CoCpCapx[x];
```

f= mol flow in [mol/s], g=metabolic rate

In[200]:=
```
Clear[fCapEcxt, fEcIsxt, fIsMyoxt, gMyoExtract];
Clear[dCFakCapxt, CFakEcxt, CFakIsxt, CFakMyoxt];
fCapEcxt[x_, t_] := PsCapEcx[x] (kCap CFakCapxt[x, t] - kEc CFakEcxt[x, t]); (* [mol/s] *)
fEcIsxt[x_, t_] := PsEcIsx[x] (kEc CFakEcxt[x, t] - kIs CFakIsxt[x, t]); (* [mol/s] *)
fIsMyoxt[x_, t_] := PsIsMyox[x] (kIs CFakIsxt[x, t] - kMyo CFakMyoxt[x, t]);(* [mol/s] *)
gMyoExtract := VCap CoFaTAr ExtractionFa / (TauCap FakMyo);
(* [mol/s] Myocyte extraction rate *)
DVkEc = kEc (VFaMemCapEc + VFaMemEcIs) / 2; (* Fa storage capacity of membrane *)
DVkIs = kIs (VFaMemEcIs + VFaMemIsMyo) / 2; (* Fa storage capacity of membrane *)
```

Out[206]=

Out[207]=

In[208]:=
```
Clear[CFakMyoxt]
EqCapC = VCap ( D[CFaTCapxt[x, t], t] +
        (ux[x] / TauCap) (D[CFaTCapxt[x, t], x] - D2nd D[CFaTCapxt[x, t], x, x] ) ) + fCapEcxt[x, t] == [illegible]
EqEcC = (VEc CoCpEcx[x] + DVkEc) D[CFakEcxt[x, t], t] - fCapEcxt[x, t] + fEcIsxt[x, t] == 0;
EqIsC = (VIs CoCpIsx[x] + DVkIs) D[CFakIsxt[x, t] , t] - fEcIsxt[x, t] + fIsMyoxt[x, t] == 0;
EqMyoC = VMy CoCpMyo D[CFakMyoxt[x, t], t] - fIsMyoxt[x, t] + gMyoExtract CFakMyoxt[x, t] == 0;

BcC = {dCFakCapxt[0, t] == 0,
   dCFakCapxt[x, 0] == 0,
   dCFakCapxt[xMax, t] == 0,
   CFakEcxt[x, 0] == 0,
   CFakIsxt[x, 0] == 0,
   CFakMyoxt[x, 0] == 0};
```

In[214]:=
```
SolC = Last[NDSolve[{EqCapC, EqEcC, EqIsC, EqMyoC, BcC},
    {dCFakCapxt, CFakEcxt, CFakIsxt, CFakMyoxt}, {x, 0, xMax}, {t, 0, tMax}]];
```

In[215]:=
```
(*Plot3D[CFaTCapxt0[x,t]/.SolC,{x,0,xMax},{t,0,20},PlotRange→All]
 Plot3D[CFaTCapxt[x,t]/.SolC,{x,0,xMax},{t,0,20},PlotRange→All]
 Plot3D[dCFaTCapxt[x,t]/.SolC,{x,0,xMax},{t,0,20},PlotRange→All]*)
```

In[216]:=
```
CFakMyot = FunctionDotProduct[Ax, (CFakMyoxt[#1, #2] /. SolC) &, {0, xMax, dx}, {0, tMax, dt}];
CFakMyoxt = Function[{x, t}, CFakMyot[t]];
BcC2 = {dCFakCapxt[0, t] == 0,
   dCFakCapxt[x, 0] == 0,
   dCFakCapxt[xMax, t] == 0,
   CFakEcxt[x, 0] == 0,
   CFakIsxt[x, 0] == 0};
```

In[219]:=
```
SolC2 = SolC;
SolC2 = Last[NDSolve[{EqCapC, EqEcC, EqIsC, BcC2},
    {dCFakCapxt, CFakEcxt, CFakIsxt}, {x, 0, xMax}, {t, 0, tMax}]];
```

In[221]:=
```
CFaTVet = FunctionDotProduct[hx, (CFaTCapxt[#1, #2] /. SolC) &,
   {0, xMax, dx}, {0, tMax, dt}];
CFaTVet2 = FunctionDotProduct[hx, (CFaTCapxt[#1, #2] /. SolC2) &,
   {0, xMax, dx}, {0, tMax, dt}];
```

In[223]:=
```
tm = 15;
FName
Show[Plot[{CFaTArt[t], CFaTVet2[t], CCpTVet[t]}, {t, 0, tm}, PlotRange → {{0, tm}, All}], ImS]
Show[LogPlot[{CFaTArt[t], CFaTVet2[t], CCpTVet[t]},
  {t, 0, tMax}, PlotRange → {10^-6, 1}], ImLogS]
```

Out[224]= 3005612.tac

Out[225]=

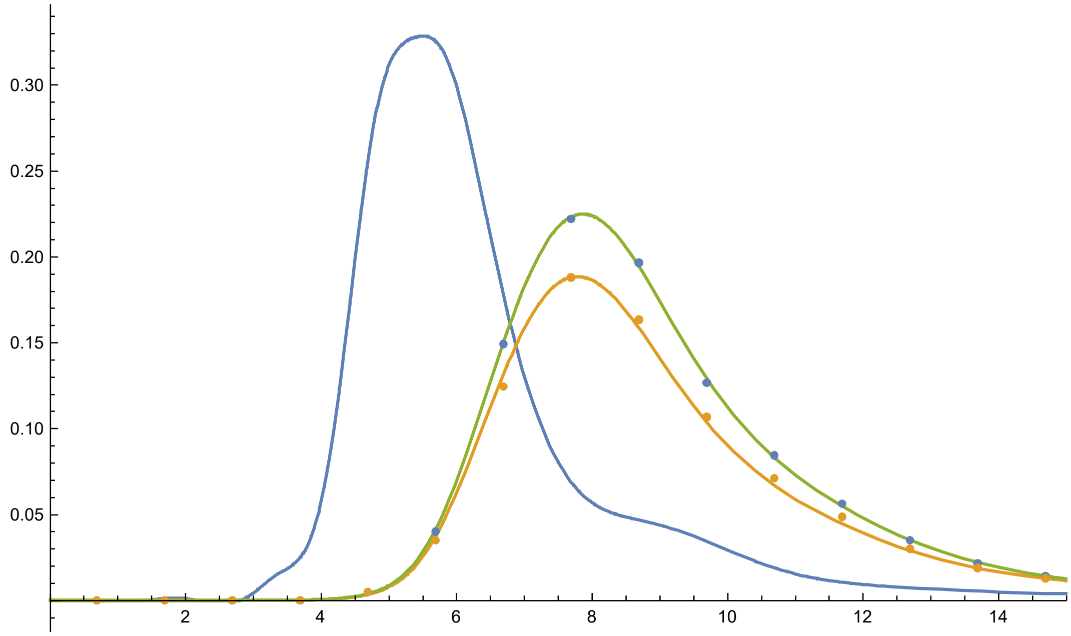

Out[226]=

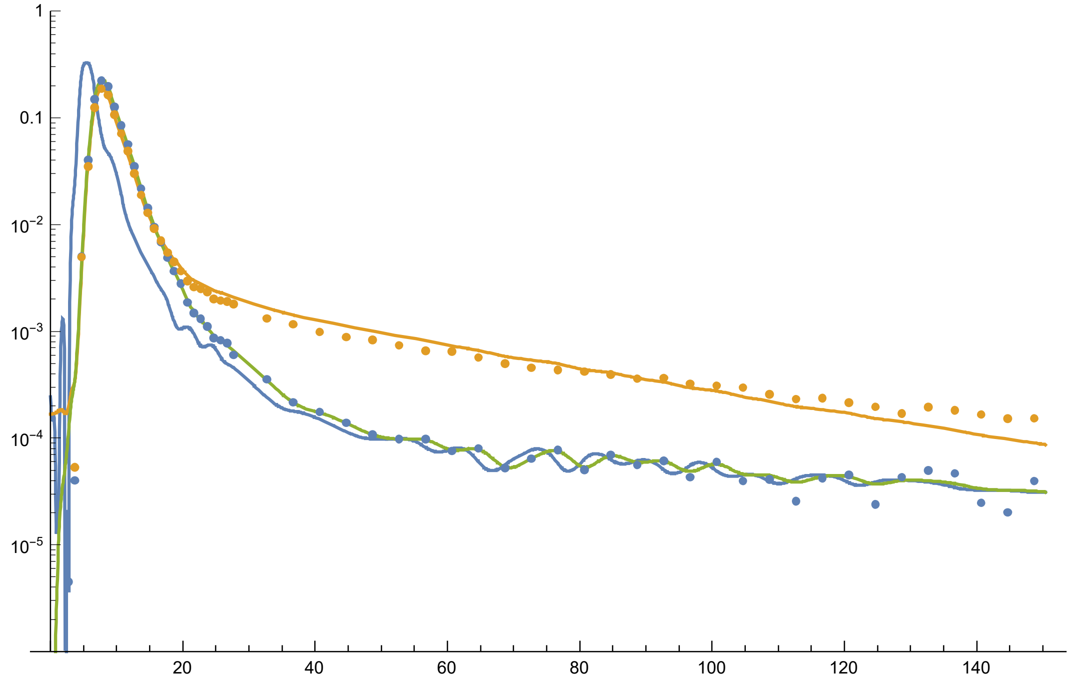

Supplement: S1 Model — This program follows the analysis as described in the manuscript, finally rendering the Fa concentration as a function of time. (PDF) [file pcbi.1004666.s003.pdf]
